# Co-expression network analyses of anthocyanin biosynthesis genes in *Ruellia* (Wild Petunias; Acanthaceae)

**DOI:** 10.1186/s12862-021-01955-x

**Published:** 2022-03-08

**Authors:** Yongbin Zhuang, Erin A. Manzitto-Tripp

**Affiliations:** 1 Department of Ecology and Evolutionary Biology, University of Colorado, UCB 334, Boulder, CO 80309 USA; 2 Museum of Natural History, University of Colorado, UCB 350, Boulder, CO 80309 USA; 3 College of Agronomy, Shandong Agricultural University, Taian, 271018 Shandong China

**Keywords:** Anthocyanin, Evolution flower color, Hybrid transcriptome-RADseq phylogeny, MYBs, Shift, Transition

## Abstract

**Background:**

Anthocyanins are major pigments contributing to flower coloration and as such knowledge of molecular architecture underlying the anthocyanin biosynthetic pathway (ABP) is key to understanding flower color diversification. To identify ABP structural genes and associated regulatory networks, we sequenced 16 transcriptomes generated from 10 species of *Ruellia* and then conducted co-expression analyses among resulting data.

**Results:**

Complete coding sequences for 12 candidate structural loci representing eight genes plus nine candidate regulatory loci were assembled. Analysis of non-synonymous/synonymous (dn/ds) mutation rates indicated all identified loci are under purifying selection, suggesting overall selection to prevent the accumulation of deleterious mutations. Additionally, upstream enzymes have lower rates of molecular evolution compared to downstream enzymes. However, site-specific tests of selection yielded evidence for positive selection at several sites, including four in *F3'H2* and five in *DFR3*, and these sites are located in protein binding regions. A species-level phylogenetic tree constructed using a newly implemented hybrid transcriptome–RADseq approach implicates several flower color transitions among the 10 species. We found evidence of both regulatory and structural mutations to *F3′5'H* in helping to explain the evolution of red flowers from purple-flowered ancestors.

**Conclusions:**

Sequence comparisons and co-expression analyses of ABP loci revealed that mutations in regulatory loci are likely to play a greater role in flower color transitions in *Ruellia* compared to mutations in underlying structural genes.

**Supplementary Information:**

The online version contains supplementary material available at 10.1186/s12862-021-01955-x.

## Background

Across angiosperms, flower color is determined by a number of different factors including the presence of one or more pigment classes (e.g., anthocyanins, non-anthocyanin flavonoids, carotenoids including xanthophylls, betalains, and chlorophyll) and environmental factors including pH and UV exposure [93, 95]. Among these different classes, anthocyanins are widely appreciated as one of the most important and evolutionarily widespread plant pigment pathways [26, 52, 72, 81]. Anthocyanins are water-soluble pigments that belong to the flavonoid biosynthetic pathway. The three primary aglycone forms of anthocyanin plus three derivatives–pelargonidin, cyanidin (including peonidin), and delphinidin (including petunidin and malvidin)–are together responsible for red, pink, and purple to blue pigmentation, respectively [92].

The Anthocyanin Biosynthetic Pathway (ABP) has been extensively studied from a molecular perspective and is highly conserved across flowering plants. Studies in model species such as petunia, snapdragon, and *Arabidopsis* have identified eight structural genes encoding the following core ABP enzymes: chalcone synthase (CHS), chalcone isomerase (CHI), flavonoid 3-hydroxylase (F3H), flavonoid 3'-hydroxylase (F3'H), flavonoid 3′5'-hydroxylase (F3′5'H), dihydroflavonol 4-reductase (DFR), anthocyanidin synthase (ANS), and UDP–3–O–glucosyltransferases (UF3GT). The temporal and spatial expression of these eight genes as well as any structural variations due to mutations in these genes, together with differential binding of pathway intermediates to enzymes depending on substrate specificity, yields flower color variation [26, 72].

Anthocyanin biosynthesis is regulated primarily at the transcriptional level by a complex of three classes of transcription factors–MYB, basic helix-loop-helix (bHLH), and WD-40 repeat (WDR) proteins–collectively referred to as the MBW complex [50]. Compared to bHLH and WDR, MYBs play a central role in recognizing specific target genes [6, 45, 94]. A plethora of studies have identified MYB homologs and their functions in various plant families. An R2R3-MYB encoding gene named *VvmybA1* is a key regulator of *UFGT* in grape skin and other parts of the grape plant [88]. In *Antirrhinum majus*, expression of *F3H*, *DFR*, *ANS* and *UF3GT* in flowers is regulated by three closely related R2R3-MYBs named *Rosea1*, *Rosea2* and *Venosa* [23, 62]. Three MYBs isolated from apple, *MdMYB1*, *MdMYB10* and *MdMYB*, and 'MYB gene *Ruby*' from *Citrus*, facilitate the accumulation of anthocyanins in the fruit [14, 20]. MYBs furthermore play tissue- and location-specific roles in flower coloration. In *Mimulus lewisii*, two R2R3-MYBs named *PELAN* and *NEGAN* regulate anthocyanin biosynthesis in petal lobes and nectar guide spots, respectively [90].

Significant progress made in elucidating ABP regulatory mechanisms in non-model plants has been possible owing to the conserved nature of ABP enzymes and their MBW regulators. However, study of ABP regulation through homolog-based approaches limits potential identification of novel, regulatory elements because most identified MYBs are highly conserved and belong to the same clade in phylogenetic analyses. Somatic mutations of identified MYBs generally affect the expression of one or a few key structural genes [17], suggesting the existence of other unidentified regulators. In the present study, in addition to identification of genetic elements involved in ABP expression through a homolog-based search method, we undertake co-expression network association analyses (Additional file 1: Fig. S1) using 16 newly generated transcriptomes from 10 closely related species of *Ruellia* (Wild Petunias: Acanthaceae) to (1) yield a first, comprehensive estimate of candidate structural and regulatory loci underlying flower coloration in *Ruellia*, (2) examine molecular evolution of these loci, (3) assess whether mutations to structural vs. regulatory elements have likely been of greater importance during flower color evolution in the group, (4) identify blocks of the ABP likely under co-regulation, and (5) identify potentially novel candidate transcription factors involved in the ABP.

## Methods

### Plant materials

This study sampled flower petal (P) and/or leaf (L) tissues from the following species, all of which are in cultivation in glasshouses at the University of Colorado–Boulder: *Ruellia bourgaei* (P), *R. breedlovei* (P), *R. brevifolia* (P), *R. elegans* (P, L), *R. fulgida* (P, L), *R. hirsuto-glandulosa* (P, L), *R. longepetiolata* (P), *R. lutea* (P, L), *R. simplex* (P, L), and *R. speciosa* (P, L). Flower petal tissues were removed from the S3 stage (Figure 3) and leaf tissues were collected at a mature stage, approximately 2–3 nodes below the uppermost meristem. All species except *R. longepetiolata* and *R. elegans* were derived from wild field populations; the former two species were acquired and grown from cuttings sent by colleagues.

### Anthocyanidin identification

We followed anthocyanidin identification and quantification methods described in Zhuang and Tripp [97]. Briefly, 25 mg of silica-dried leaf or petal tissue was placed in 1.5 ml tubes filled with 2 N HCl. Sugars were cleaved from anthocyanin molecules by placing tubes in a 103 °C heat block for 90 min then centrifuged for 5 min at 10,000 rpm at room temperature to obtain supernatants. Retained supernatants were washed twice with 400 uL of ethyl acetate and centrifuged for 1 min at 10,000 rpm to restore phase separation. The pigmented bottom layer was further washed twice with 200 uL of isoamyl alcohol and injected into an Agilent 1260 Infinity system (Thermo Scientific) for anthocyanidin identification and quantification.

### cDNA library construction and sequencing

Fresh leaf and petal tissues were collected from plants growing in the glasshouses and placed immediately into liquid N_2_ for RNA extraction. Total RNA was extracted with MasterPure™ RNA Purification Kits (Epicentre) following the manufacturer’s protocols. Integrity of total RNA was determined by running samples on a 1.2% agarose gel. RNA-seq library preparation was conducted using an Illumina ScriptSeq Complete Kit, Low Input (BL1224, Illumina) according to the manufacturer's instructions. The sequence-ready libraries were quantified using a Qubit (Invitrogen) and quality was assessed using a Bioanalyzer. Libraries were sent to the Genomics and Microarray Core, University of Colorado–Anschutz Medical Campus, then sequenced on an Illumina HiSeq2500 using 2 × 125 bp paired-end (PE) chemistry. Downstream analysis of the resulting raw data is described in Additional file 1: Fig. S1. Resulting sequences have been deposited at NCBI: http://www.ncbi.nlm.nih.gov/bioproject/PRJNA323650.

### Raw reads processing

We used Trimmomatic (Bolger et al. [9], LEADING:3 TRAILING:3 SLIDINGWINDOW:4:15 MINLEN:36’) to remove low quality bases and adaptor contaminations in our raw reads. After quality filtering, reads were then error-corrected using SEECER, a hidden Markov model (HMM)-based probabilistic error correction tool, with default parameters [34].

### Hybrid RAD loci–transcriptome approach to reconstructing a species phylogeny

To understand evolutionary relationships among the ten species used in our study, we developed a simple pipeline that uses unassembled transcriptome reads for phylogenetic reconstruction. First, all error-corrected reads were screened for the presence of the sequence tag “GAATTC”, which represents the *EcoRI* enzyme recognition sequence. The sequence tag 5'-AATTC as well as its downstream sequences were extracted. The resulting reads were used for de novo locus assembly implemented in pyRAD v3.0.6 (Eaton [19]–mindepth 15,–clustering threshold 0.85, –minsamples: 3). Resulting biallelic loci were extracted from the final alignment using an in-house Perl script. We used maximum likelihood methods to reconstruct phylogenetic relationships in RAxML v8 (Stamatakis [68],-f a; -m GTRGAMMA; -N 200).

### *Transcriptome *de novo* assembly and quality assessment*

Tissue-specific transcriptomes were assembled de novo using Trinity software release v2.2.0 [24] with a minimum contig length cutoff of 200 bp. Different parameter combinations were tested to obtain the best assembly (–min_kmer_cov: 1 or 2; –normalize_max_read_cov 30, 40 or 50; –jaccard_clip on or off). Assembly quality was evaluated using TransRate v1.0.125 [65]. Assembled transcriptomes with the best TransRate scores were selected for ORF identification using TransDecoder (https://github.com/TransDecoder/TransDecoder). Identified ORF sequences were then clustered using CD-HIT [36] to remove redundant sequences at a 99% similarity threshold. Mapping metrics were obtained using bowtie2 v2.2.6 [33] by mapping error-corrected reads back to their corresponding tissue-specific assembly. The completeness of the TransRate-selected transcriptome was assessed using BUSCO v2.0.1 (Simão [64], plant BUSCO dataset downloaded 3/31/17).

### PCA analysis

To generate a uniform read count table for downstream analysis, identified ORF sequence files from all samples were pooled together and clustered using CD-HIT-EST with a similarity threshold of 95%, and each resulting transcript was considered as a unigene then used as a reference for read mapping. Reads from each tissue-specific library were mapped to the constructed reference using bowtie2 (–sensitive), and eXpress v1.5.1 [58] was used to extract raw read counts and FPKM values for each sample. Transcripts were included in downstream analysis when > 10 reads were recovered in a minimum of five samples. Transcripts failing to meet this threshold were removed from downstream analyses. We then conducted a regularized logarithm (rlog) transformation using the rlog function in DESeq2 [39] on the remaining data. A PCA analysis was conducted on these transformed data using the plotPCA function in DESeq2 and results were visualized using the R package ggplot2 [85].

### Identification of ABP structural genes

To identify candidate structural genes involved in the ABP in all species of *Ruellia* included in this study, six core pathway genes from *Arabidopsis* (*CHS*: AT5G13930, *CHI*: AT3G55120, *F3H*: AT3G51240, *DFR*: AT5G42800, *ANS*: AT4G22880, and *UF3GT*: AT5G54060) and two branching genes from *Penstemon neomexicanus* (*F3'H*: KM388824 and *F3′5'H*: KM388828) were used as references for homolog identification using NCBI-BLAST [2], with a blast e-value threshold of 1 × 10^−100^ for both blastn and blastp, in all instances. To further improve the quality of this dataset, all identified transcripts were further blasted against NCBI's nr Database; candidate transcripts were retained only if the top blast hit was annotated as a structural gene belonging to the ABP. cDNA sequences of transcripts obtained from all species were aligned to their corresponding reference genes to assess sequence integrity using MAFFT v7.3.10 (Katoh et al. [30]; mafft–maxiterate 1000–localpair).

**Fig. 1:**
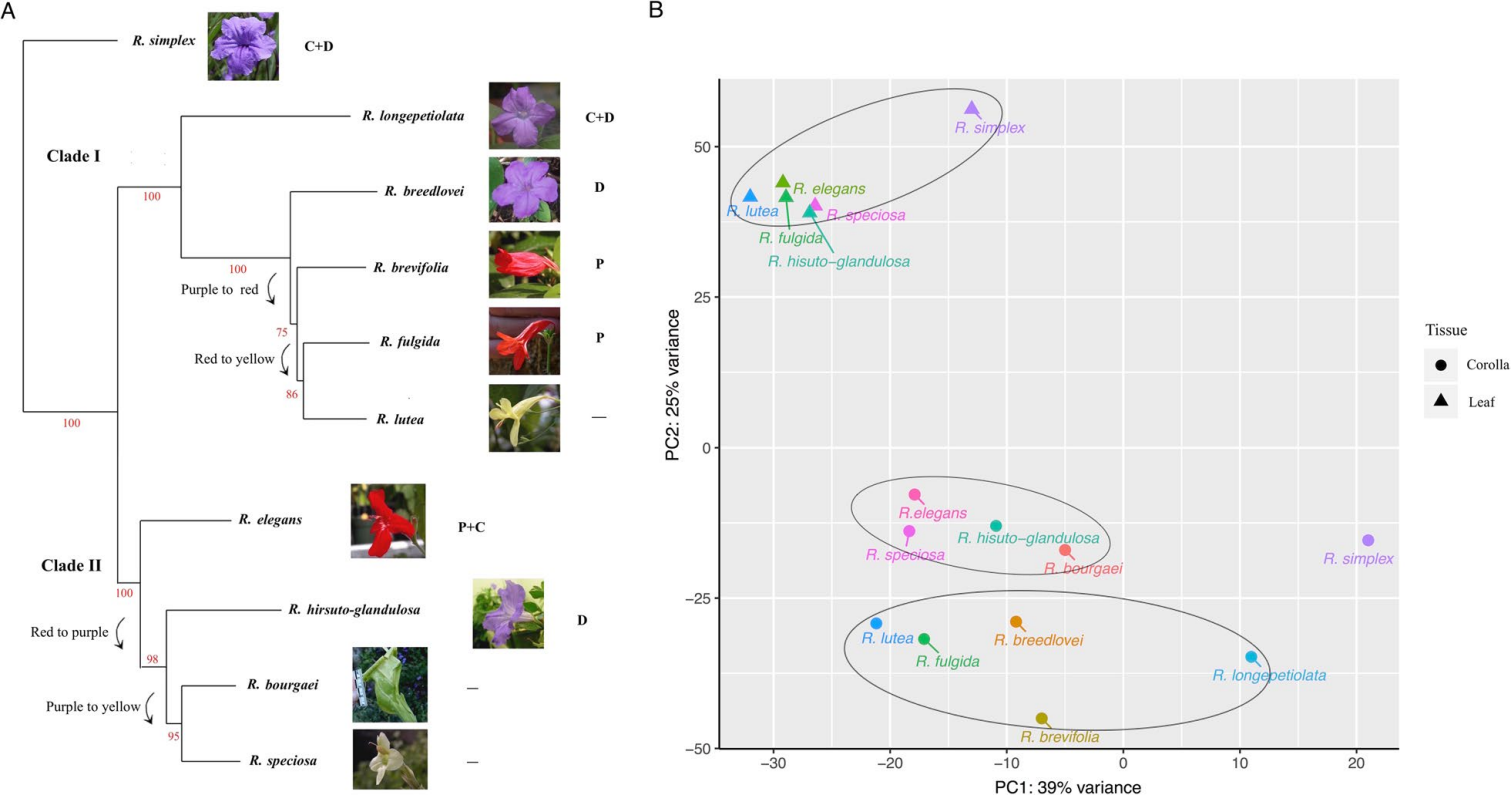
Phylogenetic tree depicting evolutionary relationships among 10 sampled species of Ruellia and principle component analysis (PCA) of transcriptome data from these species. **A**. Maximum likelihood tree was inferred using a hybrid transcriptome-RADloci approach newly implemented in this study. Briefly, raw, unassembled transcriptome reads were mined for the subset of reads that contained the GAATTC EcoR1 overhang; these yielded a total 110,263 biallelic sites following data processing in pyRAD. The ML tree was inferred using a GTRGAMMA model of sequence evolution implemented. Shown to the right of taxon labels are: photographs of flowers of the 10 study species as well as a shorthand coding of the major anthocyanin pigments present in petal tissues of these species (C = cyanidin + derivatives; D = delphinin + derivatives; P = pelargonidins+ derivatives). **B**. Gene expression profiles of ten species projected onto the first two principal components, showing two clear clusters of leaf (triangle) vs. corolla (circle) transcripts as well as two subclusters of corolla transcripts separated by PC2 that correspond to Clade I vs. Clade II in Fig. 1A

For species for which we were unable to assemble full-length cDNAs for all eight ABP loci, a second round of de novo assembly was conducted. First, all error-corrected reads were mapped to the reference gene of its most closely related species (see Fig. 1A) using bowtie2 v2.2.6 (Langmead and Salzberg [33],–sensitive). All mapped reads were then extracted using samtools v1.4.1 [35] and served as input for de novo assembly in SPAdes v3.9.0 [5] with default parameters.

### Identification of conserved MYBs, bHLH and WDR

To identify candidate ABP regulatory MYB, bHLH, and WDR-type transcription factors, all protein sequences translated by TransDecoder were subject to domain searching for the MYB domain (PS51294), bHLH domain (PS50888) and WD-40 repeat (PS50294) using ps_scan [22] with default parameters. For MYB domain-containing proteins, the number of MYB domains was counted using an in-house Perl script; we retained only R2R3-type MYBs for further analysis. Seven MYBs with a known or candidate role in regulating the ABP were used as references during searches for homologs in *Ruellia* using NCBI blastp [2] with with a blast e-value threshold of 1 × 10^−100^. These included eight R2R3-type MYB proteins: PAP1 (AtMYB75, AT1G56650), PAP2 (AtMYB90, AT1G66390), mgv1a023671m, mgv1a024996m, mgv1a019326m, mgv1a025765m, 10bHLH proteins: AtbHLH34 (AT3G23210), GL3 (AT5G41315), EGL3 (AT1G63650), TT8 (AT4G09820), AtbHLH32 (AT3G25710) MYC3 (AT5G46760), MYC4 (AT4G17880), Migut.A00733.1 Migut.D00287.1 Migut.E01090.1; and two WDR proteins: TTG1 (AT5G24520) and ATAN11 (AT1G12910).

### Co-expression analysis of candidate ABP genes

To evaluate the expression correlations among identified candidate ABP genes, their FPKM values were used for testing the significance of correlations (an FDR adjusted p-value threshold of < 0.05) using the corr.test() function in the R package “psych” [56] and p-values were adjusted using the Holm-Bonferroni method. All statistical analyses were conducted within the R environment. The correlation was visualized as a matrix using the R package “corrplot” [80]. For candidate ABP structural genes associated with *MYB10L1*, correlations were predicted using a linear model with a 95% confidence level interval (geom_smooth(method = "lm", level = 0.95)) and visualized using ggplot2 [85].

### Non-synonymous to synonymous mutation rates and tests for selection

To test for evidence of positive selection on ABP genes, MAFFT-aligned cDNA sequences and their corresponding coding sequences were used to guide coding sequence (CDS) alignments in Pal2Nal (Suyama et al. [69], http://www.bork.embl.de/pal2nal/). The ratio of non-synonymous to synonymous mutations (dN/dS, or ω) was estimated using codon-based maximum likelihood methods implemented in codeml, a module in the PAML package (version 4.8; Yang [89]). To test for positive selection across an entire gene, we used the M0 model, which assumes the same *ω* ratio for all sites in the gene. To test for positive selection in a site-specific manner (for nuances and best practices of site-specific dN/dS analyses, see, for example, [8, 67, 78), we first identified sites under positive selection through Naive Empirical Bayes (NEB) analysis (P > 95%) using the site models M1, M2, M7 and M8, which allow the *ω* ratio to vary among sites. Models M1 + M2 and M7 + M8 form two pairs of models that can be used to test for positive selection using a likelihood ratio test (LRT). Model M1and M2 allow the site classes ω = 1 and 0 < ω < 1, and model M2 additionally includes a third class: ω > 1 [89]. Models M7 and M8 include several site classes with ω ratios that follow a beta distribution, and model M8 additionally includes a ω > 1 class. Two sets of model comparisons were conducted for each gene: the first compares M1/M2 whereas the second compares M7/M8. Codon sites that are under positive selection were identified using the Bayes Empirical Bayes (BEB) method [89].

### Secondary structure analysis and functional predictions

To examine potential effects of amino substitutions on protein function, we predicted the secondary structure of proteins as well as functional effects of mutations at amino acid sites under positive selection. Secondary structure of proteins (helices, sheets, and coils) was predicted using the PredictProtein online server (Yachdav et al. [87], https://www.predictprotein.org, last accessed 07/21/2017). Functional effects of sequence variants were predicted using snap2, which is integrated into the predictProtein server [87].

### Identification of ABP interactive pathways through co-expression network analysis

To test for correlations in expression among assembled transcripts and ABP genes, transcripts with at least 10 read counts and transcripts that were found in at least three samples were selected. Pearson correlation coefficients were calculated using the corr.test function in the R package “physic” [56]. To calculate adjusted P values, expression levels of each ABP gene were permuted 1000 times and calculated as (*r* + 1)/(*n* + 1), where *n* = 1000 and *r* is the number of these replicates that produce a test statistic greater than or equal to that calculated for the actual data [47]. We deemed correlations to be significant when Pearson’s *r* ≥ 0.65 and *P* < 0.05. All transcripts with expression levels significantly associated with the 12 identified candidate structural genes were annotated using Trinotate pipeline release version 3.0.0 (Grabherr et al. [24], http://trinotate.sourceforge.net/) with a blast e-value threshold of 1 × 10^−5^. We additionally built a custom plant transcription factor database for ABP loci using data downloaded from the Plant Transcription Factor Database v4.0 (Pérez-Rodríguez et al. [51]; http://planttfdb.cbi.pku.edu.cn, last accessed 04/18/2017). KEGG pathway analysis was conducted on all resulting transcripts using BlastKOALA (Kanehisa et al. [29], http://www.kegg.jp/blastkoala/ Last accessed 4/01/2017) and was further processed using an in-house Perl script then visualized using the R package ggplot2 [85].

### Phylogenetic analysis and sequence comparison

We aligned translated protein sequences of ABP genes using MAFFT v7.3.10 (Katoh et al. [30]; mafft–maxiterate 1000–localpair) and then identified conserved regions of resulting alignments using Gblocks [70]. Phylogenetic analyses were conducted on final alignments using RAxML and the GTRGAMMA substitution model, which is more computationally intensive compared to GTRCAT but is suitable for datasets with smaller numbers of taxa, such as ours (see RAxML documentation: https://cme.h-its.org/exelixis/resource/download/NewManual.pdf) with 100 bootstrap replicates. Resulting phylogenetic trees were viewed using Evolview (Zhang et al. [91], http://www.evolgenius.info/evolview, last accessed 3/21/2017).

### Real time qPCR

To confirm s transcript’s abundance measured from the RNA-Seq data and better characterize assembled homologs of *F3'H* and *DFR*, we extracted total RNA from petal tissues of *Ruellia simplex* collected at different growth stages using MasterPure™ RNA Purification Kit (Epicentre) following the manufacturer’s protocols. For reverse transcription, ~ 500 ng of DNase I-treated total RNA was used for cDNA synthesis using the GoScript™ Reverse Transcriptase system (Promega) with the oligo(dT)_15_ primer. qPCR was carried out using SYBR green I based master Mix (Roche Life Science, Indianapolis, IN) with 2 min at 95 °C, 40 cycles of 20 s at 95 °C, 45 s at 57 °C, and 30 s at 72 °C, and then 5 min at 72 °C (Applied Biosystems). For gene expression analysis, the 18 s reference gene was used as an internal control to normalize *Ct* values. For each sample, three technical replicates were conducted.

## Results

### HPLC analysis of anthocyanin accumulation in leaf and petal tissues

To phenotype ABP pigments that underlie flower color variation in *Ruellia*, 10 species with distinct petal colors (four purple, three red, and three yellow) were selected based on visual inspection coupled with floral color delimitation into one of several discrete categories. The latter was accomplished via floral reflectance measured using an Ocean Optics JAZ-COMBO Spectrometer (Fig. 1A; Additional file 15: Data S1). Presence/absence of cyanidin, delphinidin, pelargonidin and their derivatives were measured in both petal and leaf tissues using HPLC (Additional file 16: Data S2). No anthocyanins were detected in petal tissues of the three yellow-flowered species (*Ruellia bourgaei, R. speciosa*, and *R. lutea*)*.* Two of the three red-flowered species (*R. brevifolia*, *R. fulgida*) produced only pelargonidin whereas in the third red-flowered species (*R. elegans*), both pelargonidin and cyanidin were detected. Two of the purple-flowered species (*R. breedlovei*, *R. hirsuto-glandulosa*) produced only delphinidins whereas the remaining two purpled-flowered species (*R. simplex*, *R. longepetiolata*) manufactured both delphinidins and cyanidins (Fig. 1A, Additional file 16: Data S2). No species in this dataset produced the combination of pelargonidin and delphinidin pigments. In leaf tissues, cyanidins were found in all species except *R. bourgaei* and *R. hirsuto-glandulosa* (Additional file 16: Data S2).

### De novo assembly of tissue-specific Ruellia transcriptomes

To help elucidate molecular mechanisms regulating anthocyanin accumulation, we analyzed data from 12 newly sequenced transcriptome libraries (eight from flower petals, four from leaf tissues) together with data from four previously published libraries (two from flower petals, two from leaf tissues; [97]. Quality statistics for tissue-specific assemblies using the best TransRate score are shown in Table 1. On average, 165.860 ± 28.003 transcripts were assembled with an average N50 of 1611 ± 194 nt, and similar GC contents were found across all libraries (42.50 ± 0.53%). TransDecoder identified 73.614 ± 18.417 Open Reading Frames (ORFs) in assembled transcriptomes. Representativeness of each assembly was evaluated by mapping error-corrected reads back to the assembly. A high mapping rate (95.96 ± 2.10%) was obtained across all assemblies and among mapped reads: nearly all (98.78 ± 1.04%) could be mapped concordantly as read pairs. We also evaluated transcriptome completeness by searching for single-copy orthologs of highly conserved plant genes in each assembly using BUSCO v2 [64]. On average, assembled transcriptomes, regardless of whether they derived from petal or leaf tissue, contained a high percentage (74.06 ± 5.91%) of the 1440 groups of conserved plant genes deposited in the BUSCO database; this high percentage further reflects the completeness of our transcriptome assemblies.

**Table 1:** Summary statistics of assembled *Ruellia* transcriptomes

Species	Tissue	# of transcripts	N50 (nt)	GC (%)	# of ORF	BUSCO (%)	Mapping rate (%)
*R. bourgaei*	Petal	143979	1613	43.41	84262	82.60	97.69
*R. lutea*	Petal	202066	1782	42.11	72821	76.67	96.78
*R. speciosa*	Petal	183957	1353	42.66	53524	68.67	90.99
*R. fulgida*	Petal	202811	1911	41.85	82087	78.40	96.72
*R. elegans*	Petal	155972	1313	42.71	57097	65.76	93.43
*R. brevifolia*	Petal	171861	1909	42.68	99328	76.18	98.06
*R. breedlovei*	Petal	162669	1418	42.61	54982	63.82	92.84
*R. hirsuto-glandulosa*	Petal	174679	1690	42.10	64848	73.40	94.57
*R. longepetiolata*	Petal	103568	1718	43.27	62315	62.56	97.08
*R. simplex*	Petal	187555	1769	42.67	107402	69.44	97.27
*R. lutea*	Leaf	149699	1741	41.99	70772	76.25	98.03
*R. speciosa*	Leaf	141301	1308	42.06	54952	75.48	94.47
*R. fulgida*	Leaf	125607	1551	42.62	57570	75.00	97.84
*R. elegans*	Leaf	195570	1522	41.68	76339	73.76	95.91
*R. hirsuto-glandulosa*	Leaf	168329	1626	42.17	70680	76.25	96.63
*R. simplex*	Leaf	184144	1563	43.35	108853	80.69	97.05
Mean		165860	1611	42.50	73614	74.06	95.96
SD		28003	194	0.53	18417	5.91	2.10

### Reconstruction of phylogenetic relationships in Ruellia

To resolve phylogenetic relationships among the 10 species of *Ruellia*, we employed a new approach that involves retrieving RAD loci from transcriptome data. Phylogenies constructed using assembled transcriptome data sometimes face challenges due to complications associated with pseudogenes and paralogous loci, resulting in potentially misassembled contigs [82]. We thus implemented a hybrid transcriptome–RADseq phylogenetic tree building approach that made use of a subset of error-corrected reads having the *EcoR1* GAATTC overhang. These reads were treated in a manner similar to RADtags and were analyzed using pyRAD [19]. The minimum locus length was set to 35, but the resulting average read length was 78nt, which is suitable for phylogenetic analysis as it has been shown that large phylogenetic tree can be built accurately from even short (50 bp average) sequences [27]. A total of 110,263 loci were concatenated and used for phylogenetic inference via maximum likelihood implemented in RAxML v8 [68]. Branch support as assessed via 100 bootstrap replicates was ≥ 75% for all nodes (most nodes with ≥ 95% support), and the resulting phylogeny composed of two major clades (Fig. 1A) mirrors relationships recovered in prior phylogenetic studies in the genus [75]. We specifically did not conduct ancestral state reconstructions of flower color and estimations of color transitions due to very few species terminals [59], especially in a taxonomically species-rich genus such as *Ruellia* (ca. 350 species worldwide). However, the 10-tip phylogeny suggests four potential color transitions: in Clade I, one from purple to red and one from red to yellow flowers, and in Clade II, one from red to purple and one from purple to yellow flowers.

### PCA analysis of leaf and petal transcriptomes

To assess overall expression level similarities among transcriptomes, we conducted a principal component analysis (PCA) on normalized gene count data based on combined petal and leaf transcriptome data from *R. simplex*, which had the best assembly statistics among all species (Table 1). After removing transcripts with low expression levels (see “Methods”), a total of 60,626 transcripts were used in PCA analysis. Results demonstrate that flower petal vs. leaf transcripts were separated into two clusters (Fig. 1B). Additionally, two subclusters among the flower petal transcripts can be identified and correspond to the two clades resolved in our phylogeny (Fig. 1A): Clade I (*R. elegans*, *R. speciosa*, *R. hisuto-glandulosa*, *R. bourgaei*) and Clade II (*R. lutea, R. fulgida*, *R. breedlovei*, *R. brevifolia*).

### Identification and co-expression analysis of candidate ABP structural genes

**Fig. 2:**
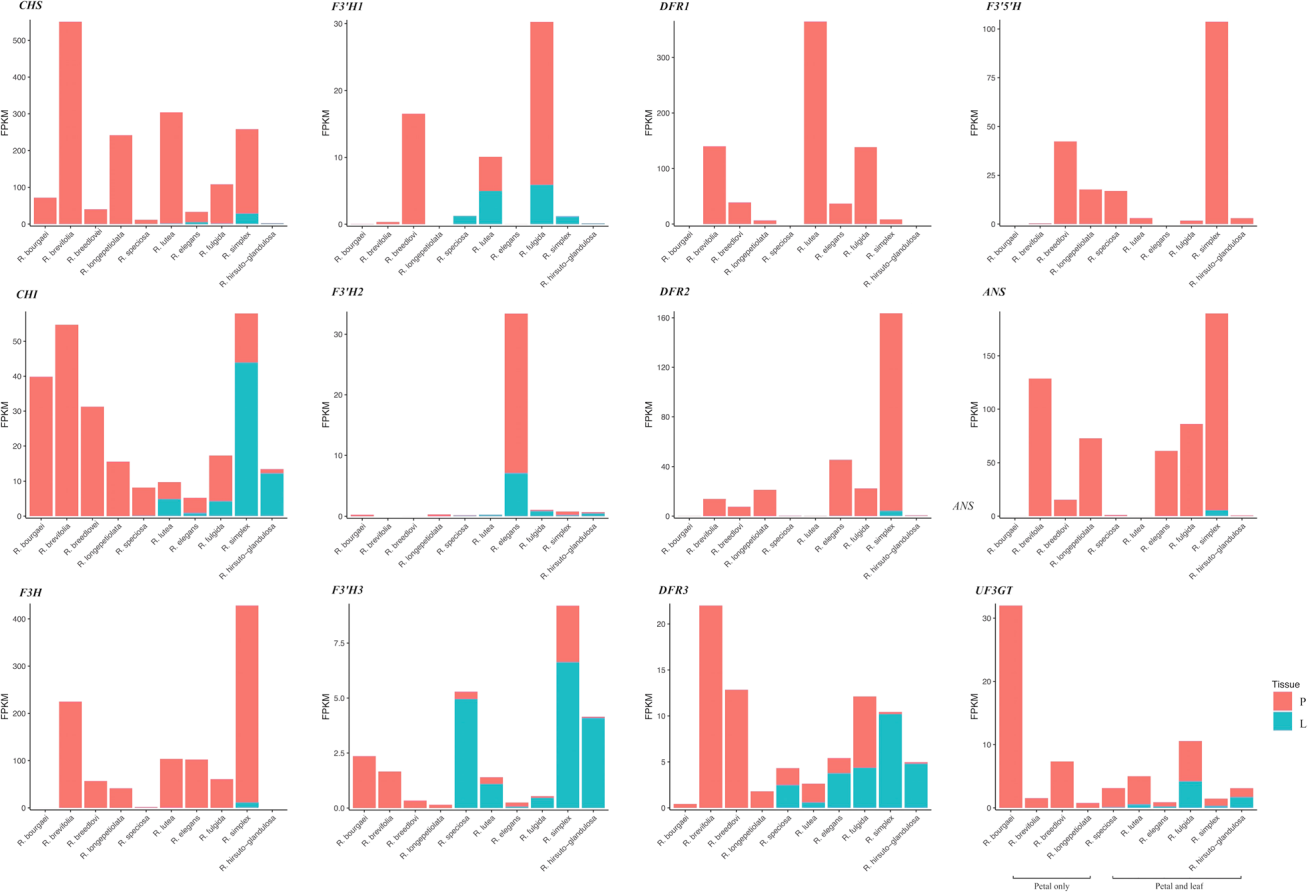
Transcript abundance of candidate anthocyanin structural genes identified in this study; both corolla and leaf transcriptome data were sequenced for six of the ten species of *R. speciosa, R. elegans, R. fulgida, R. simplex* and *R. hirsute-glandulosa*. Shown are petal (red) and leaf (cyan) transcript levels for several genes found as a single copy in among all samples (*CHS, CHI, F3H, F3'5'H, ANS, UF3GT*) as well as two genes found with three copies each (*F3'H* [copies 1–3] and *DFR* [copies 1–3])

Our BLAST + search of Trinity-assembled *Ruellia* transcriptomes against known ABP genes identified 12 orthologous candidate ABP structural genes in *Ruellia*. Among these, one copy each of *CHS*, *CHI*, *F3H*, *F3′5'H*, *ANS* and *UF3GT* was identified whereas three copies each of *F3'H* and *DFR* were recovered. Subsequent reference-based gene assembly using SPAdes v3.10.1 [5] recovered full-length cDNA of ABP genes in most of the species (Additional file 12: Table S1, Additional file 2: Fig. S2). We then measured expression levels of the 12 candidate genes in FPKM (fragments per kilobase of transcript per million fragments sequenced) and compared results in a tissue-specific manner. Results indicate that expression of *DFR1* and *F3′5'H* was restricted to petal tissues whereas expression of all other genes occurred in one or more leaf samples (Fig. 2).

**Fig. 3:**
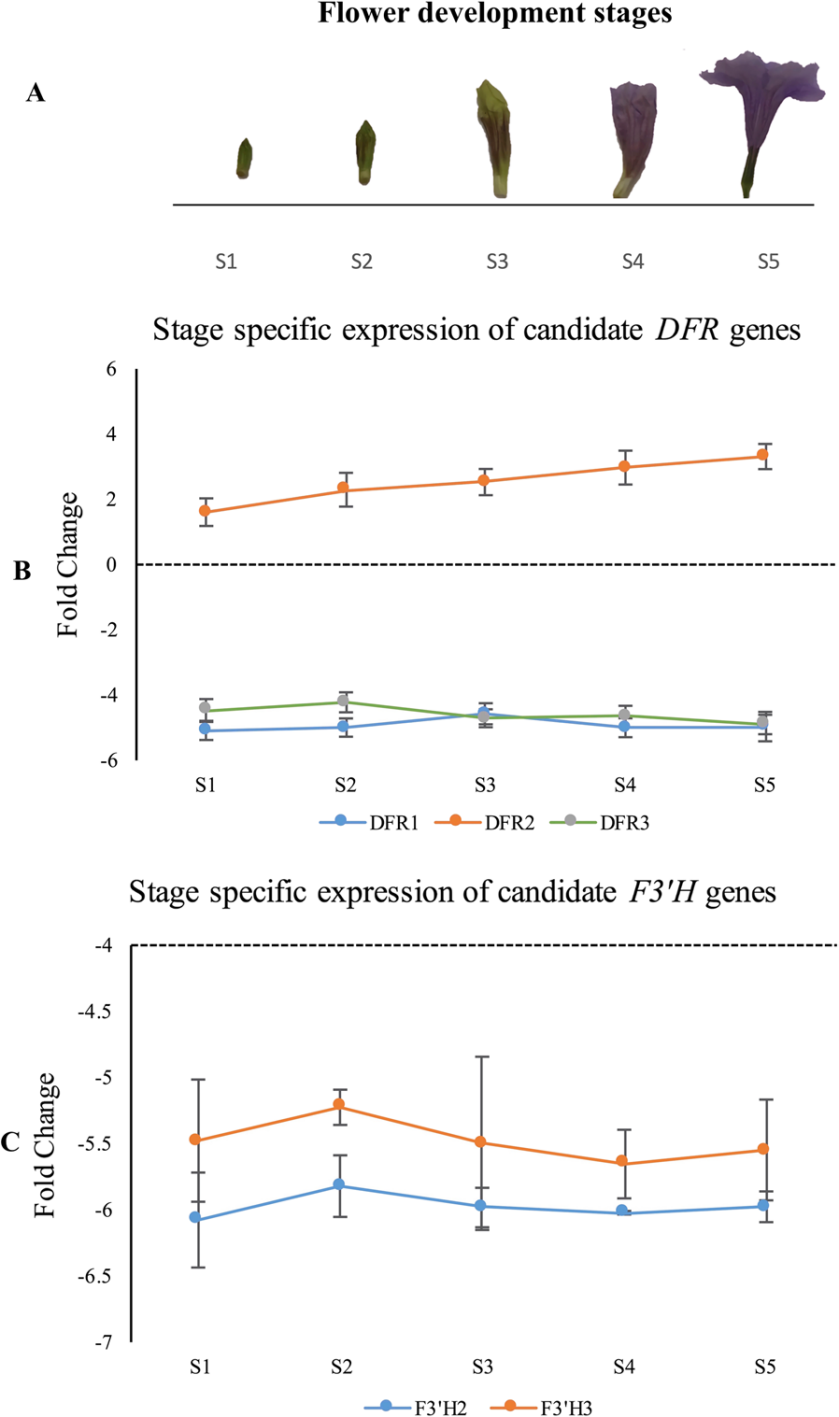
Relative expression of *F3'H* and *DFR* copies in Ruellia simplex during flower development based on qRT-PCR. Relative expression levels were calculated based on transcript abundance of the 18S gene in Ruellia using formula: Ct(18s)-Ct(gene). **A**. Five stages of floral development, from bud to fully anthetic flower. **B**. Stage-specific expression of *DFR*. **C**. Stage-specific expression of *F3'H* (note that *F3'H1* was not expressed in *R. simplex*). Results show that *DFR3* as well as *F3'H2* and *F3'H3* peak in expression levels during stage 2, suggesting possible coordinated regulation of these three loci

We further characterized the multiple copies of *F3'H* and *DFR* identified among our *Ruellia* transcriptomes by comparing translated amino acid sequence across samples (Additional file 2: Fig. S2) as well as expression patterns measured at five flower developmental stages in *Ruellia simplex* using transcript-specific primers and qPCR (Fig. 3, Additional files 13, 14: Table S2, S3). Two of the three *F3'H* copies (*F3'H1*, *F3'H2*) were highly homologous (sequence divergence: 23.44% across all samples; Additional file 2: Fig. S2) whereas *F3'H3* was markedly divergent from the former two (Additional file 2: Fig. S2). Similarly, *DFR3* is highly divergent from the other two copies (i.e., *DFR1* and *DFR2*). This marked divergence in *F3'H3* and *DFR3* coupled with more widespread expression of these two copies in leaf tissues across species (compared to expression of *F3'H1*, *DFR1*, *DFR2*, and to a lesser extent *F3'H2* in petal tissues; Fig. 2) indicates potential functional diversity of these two enzymes.

Using qRT-PCR, developmental stage-specific expression of multiple copies of *F3'H* and *DFR* found in *R. simplex* petals (i.e., *DFR1, DFR2, DFR3* and *F3'H2* and *F3'H3* [*F3'H1* not expressed in petals of this species; Fig. 2]) confirmed transcriptome-based expression patterns of ABP enzymes in this species (Fig. 3). These data further support the conclusion that our transcriptome assemblies are relatively complete and are of high quality. Analyses of *R. simplex* indicated that expression of *F3'H2*, *F3'H3* and *DFR3* all peaked at stage two of five developmental stages, also indicating potentially coordinated regulation of these three genes (Fig. 3).

**Fig. 4:**
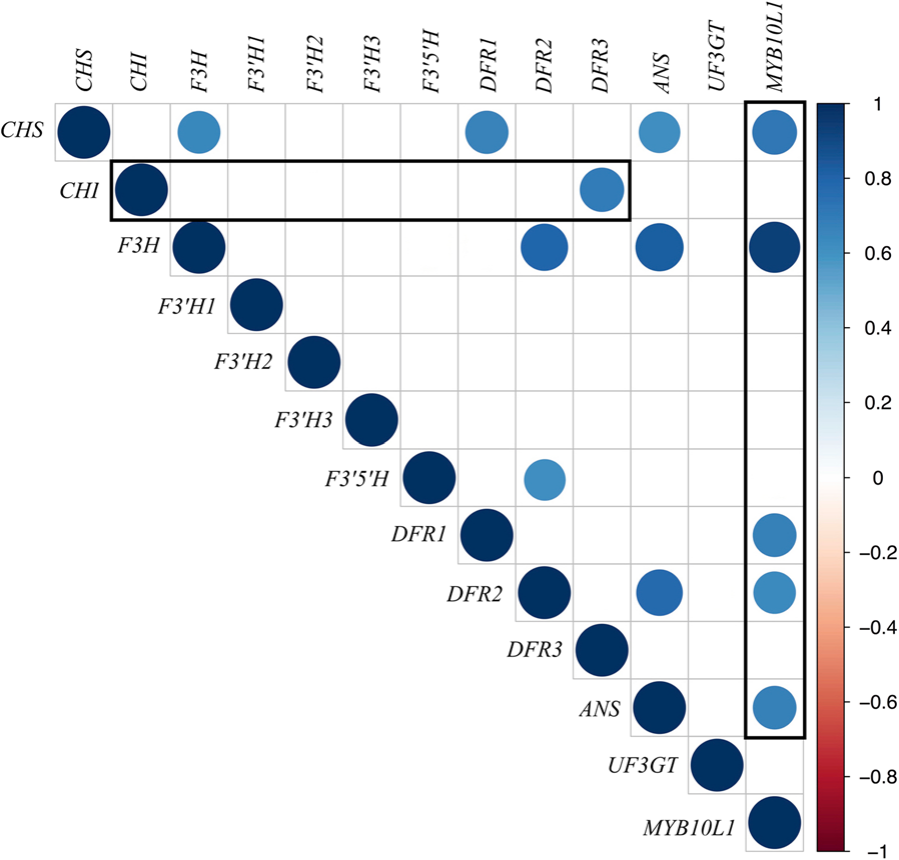
Correlations among 13 conserved ABP-associated loci using the corrplot package in R (r ≥ 0.65). Predicted regulation blocks were highlight

Network based co-expression analysis across tissue types in all samples recovered strong, correlated expression among *CHS*, *F3H*, *DFR1*, and *ANS* as well as between *CHI* and *DFR3* (r ≥ 0.65), indicating possible blocks of structural genes under regulatory control (Fig. 4). Taken together, results indicate that regulation of ABP is highly coordinated and likely involves different regulatory blocks.

### Identification and co-expression analysis of candidate ABP regulatory genes

**Fig. 5:**
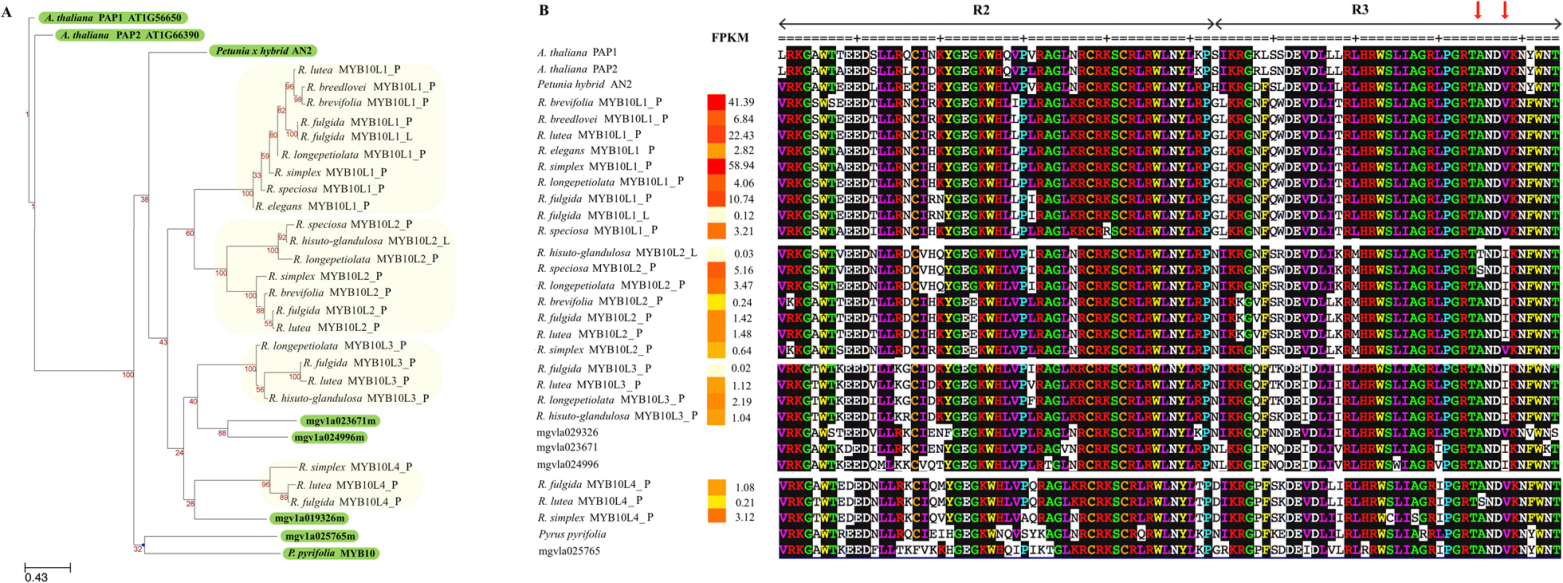
**A**. Phylogenetic analysis of candidate ABP MYB regulators identified in Ruellia petal transcripts plus orthologs that have been functionally validated to regulate the ABP in other flowering plant species (Arabidopsis thaliana, Petunia x hybrida, and Pyrus pyrifolia, highlighted in green; four MYB orthologs from Mimulus guttatus were also included in phylogenetic analyses given the close evolutionary relationship of Mimulus to Ruellia, but these four copies have not been functionally validated in prior works). Four, strongly supported clades of candidate MYBs in Ruellia were recovered (highlighted in yellow). **B**. Protein sequence alignment of candidate ABP MYB regulators in Ruellia and orthologs in other species. Relative expression of each gene shown in FPKM (fragments per kilobase of transcript per million fragments sequenced). Results indicate that copies from the MYB10L1 clade in Ruellia were among the most highly expressed and also contain an amino acid residue ('ANDV') that has been reported to characterize MYBs that regulate the ABP (see text); this residue was also found copies from the MYB10L4 clade, but transcripts in this clade were in low abundance. In contrast, a non-synonymous substitution yielding an 'ANDI' residue characterized most of the candidate Ruellia MYBs in the MYB10L2 and MYB10L3 clades, and this residue is conserved across MYBs that function elsewhere besides in ABP production in other plant families

Our homology-based searches for candidate MBW regulatory elements in *Ruellia* recovered four clades of MYBs (Fig. 5), two of WDRs (Additional file 3: Fig. S3), and three of bHLHs (Additional file 4: Fig. S4). Transcript expression indicated that the three families of transcription factors exhibited different expression patterns: expression of all four clades of MYBs was specific to petals (Fig. 5) whereas WDRs and bHLHs were expressed in both petal and leaf tissues (Additional files 3, 4: Figs. S3 and S4). Of the four MYB clades, copies of *MYB10L1* were relatively highly expressed compared to copies in the *MYB10L2*, *MYB10L3*, and *MYB10L4* clades (Fig. 5B). *MYB10L3* and *MYB10L4* were expressed only in a few species and at relatively low levels (Fig. 5).

Network based co-expression analysis across tissue types in all samples recovered strong, correlated expression of *MYB10L1* with five candidate structural genes (*CHS, F3H, DFR1, DFR2* and *ANS*; r ≥ 0.65; Fig. 4) and a weak correlation with *F3′5'H* (r = 0.56). No correlations among expression of *MYB10L2*, WDRs, bHLHs, and candidate structural genes were found; co-expression analyses were not conducted for *MYB10L3* and *MYB10L4* due to limited data.

We compared amino acid-predicted sequences of MYBs from our four identified clades in *Ruellia* to amino acid translations of functionally validated MYBs known to regulate ABP in other flowering plants: *Arabidopsis thaliana*, *Petunia* x *hybrida*, *Pyrus pyrifolia*, and *Mimulus guttatus*. Specific attention was given to the amino acid sequence residue 'ANDV' because this residue has been reported to distinguish anthocyanin from non-anthocyanin MYBs [37]. We found this residue to be present in candidate *Ruellia* MYBs from the MYB10L1 and MYB10L4 clades (Fig. 5B; note that expression levels of *MYB10L4* copies were markedly lower than were expression levels of *MYB10L1* copies). In contrast, replacement of the amino acid V with an I (resulting in the residue 'ANDI' and considered to be a conserved signature for non-anthocyanin gene regulation) characterized most of the *MYB10L2*s and *MYB10L3*s (as well as two of the four *Mimulus guttatus* sequences; Fig. 5B). We detected an additional non-synonymous mutation in this residue in *Ruellia lutea*, which resulted in an A to an S amino acid substitution (yielding 'SNDV'; Fig. 5B).

### Estimating amino acid substitutions in ABP loci and potential effects on protein function

**Fig. 6:**
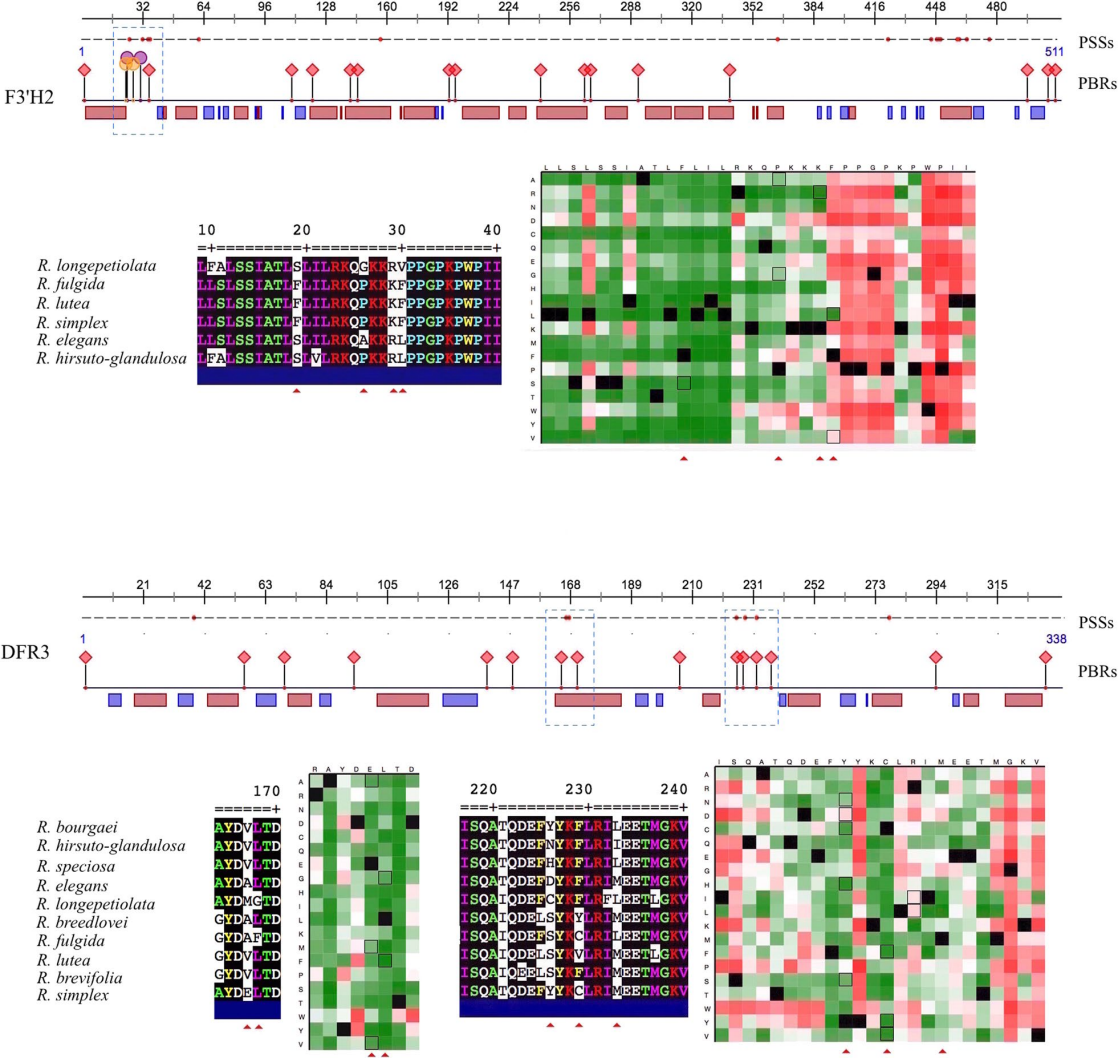
Detection of positive selection on several amino acid sites in F3'H2 and DFR3 in Ruellia and predicted effects on protein function. Upper Panels (both genes). Amino acid alignment positions shown at top. Black, dashed line with red dots denote sites under positive selection based on analyses implementing a M1 + M2 and M7 + M8 model of evolution. Protein binding regions (red diamonds) containing sites under positive selection marked with blue frames. yellow/orange circles stand for predicted nucleotide binding region, and the pink and blue rectangles stand for helex and strand respectively. Lower Panels (both genes). Expanded, view of blue frames showing amino acid substitutions and sites under positive selection, these marked by red triangles at bottom of alignment. To the right, predication of functional effects of sequence variants conducted using R. simplex as a reference. Sites under positive selection marked by red triangles and highlighted with a black outline. Dark red (scores > 50): strong signal for a potential functional effect; white (scores < 50 or > −50): weak signal for potential effect; green (scores < −50): neutral or no effect

To test whether codon sites of identified ABP loci have potentially undergone adaptive evolution, we conducted tests of non-synonymous to synonymous substitution rates (dN/dS ratios, or *ω*) across all candidate structural genes plus one candidate MYB predicted to be significantly associated with the ABP in *Ruellia* (see Fig. 4). We used genomic data from the closely related species *Ruellia matudae* to serve as an outgroup for dN/dS analyses. First, we implemented the site test model M0 in codeml, which assumes a single *ω* ratio across all sites in an alignment. Results yielded dN/dS ratios that ranged between 0.20 and 0.72 (Table 2). Among all loci tested, *DFR3* had the highest dN/dS ratio (0.72) followed by *F3'H2* (0.54) and *MYB10L* (0.52). The fact that no gene was found to have dN/dS ratio > 1 indicates that all ABP genes are predominantly under purifying selection. However, because positive selection is unlikely to affect all sites in a given protein over extended evolutionary time, we further explored the potential of positive selection on individual amino acid sites. Results indicated that several sites in both *DFR3* and *F3’H2* are likely to be under positive selection (Fig. 6). Subsequent protein secondary structure analysis indicated that four of the sites that appear to be under positive selection in *F3’H2* and five sites in *DFR3* are located inside protein binding regions (PBRs).

**Table 2:** Table showing dN/dS ratios of candidate ABP genes in *Ruellia*

	*CHS*	*CHI*	*F3H*	*F3'H1*	*F3'H2*	*F3'H3*	*F3′5'H*	*DFR1*	*DFR2*	*DFR3*	*ANS*	*UF3GT*	*MYB10L*
dN	0.07	0.07	0.11	0.08	0.17	0.1	0.06	0.06	0.04	0.14	0.11	0.08	0.17
dS	0.25	0.28	0.25	0.17	0.32	0.27	0.19	0.18	0.2	0.2	0.38	0.22	0.33
dN/dS	0.29	0.25	0.45	0.5	0.54	0.38	0.31	0.36	0.2	0.72	0.29	0.36	0.52

We further examined the potential effects of amino acid substitutions on protein function at the above sites likely to be under positive selection. Using algorithms that predict both secondary structure and functional effects, we found one substitution in F3'H2 and three substitutions in DFR3 for which a moderate functional effect of the mutation is predicted (colored as pink in Fig. 6). These results indicate that, at these sites, observed amino acid substitutions are more likely to influence the stability or substrate binding affinity of the resulting protein or even cause protein dysfunction. At all other sites, observed amino acid substitutions colored as green indicate a strong signal for neutral effect.

### Co-expression analysis of transcripts and associations with ABP loci

After removing transcripts that either had low expression levels or were expressed only in a small fraction of our study species (i.e., fewer than 3), a total of 60,613 transcripts were tested for correlated expression with the 13 ABP structural genes identified in *Ruellia*. Significant positive correlations were found for 1748 transcripts (2.89% of total transcripts present in 3 or more species, Pearson’s *r* ≥ 0.65, *P* < 0.05). We used the KEGG classification system to predict and annotate functionality of these ABP-associated transcripts by assigning them to known biochemical pathways. Of the 1748 transcripts, 532 (30.43%) were assigned KEGG annotations. Among these, genes involved in a variety of metabolic pathways formed the largest group (Additional files 5, 6: Figs. S5 and S6), among which 10 are putatively involved in phenylpropanoid biosynthesis and two are involved in flavone and flavone biosynthesis.

To gain further insight into interactions between transcripts and ABP loci as well as identify potential novel regulatory elements, transcription factors were further characterized using a custom database derived from the Plant Transcription Factor Database. We identified 13 families of TFs that are positively correlated to ABP loci expression in *Ruellia* (Additional file 5: Fig. S5). Among these, MYBs were the dominant group of ABP-associated TFs. Among 19 specific MYBs identified, nine belonged to the R2R3 family including *MYB10L1* that we earlier identified as likely to be especially relevant to ABP expression in *Ruellia* based on homology to functionally verified MYBs in other plant families (Fig. 5). To further characterize potential function roles of the remaining eight R2R3 MYBs, we used amino acid sequences (derived from *R. simplex*) in blast searches against the NCBI nr database as well as in phylogenetic analysis with R2R3 MYBs from *A. thaliana* (Additional file 7: Fig. S7). Function annotations of these R2R3 MYBs suggest regulatory roles in either anthocyanin biosynthesis (*RsMYB10L1*, *RsMYB10L2*, *RsMYB10L3*, *RsMYB10L4* and *RsMYB114L*, homologs of *AtMYB75*, and *AtMYB114*; *RsMYB305L1* and *RsMYB305L2*, homologs of *AtMYB57*, *NtMYB305*; [10, 38]) or lignin biosynthesis (*RsMYB46*, homolog of *AtMYB46*,*RsMYB306L*, homologs of *AtMYB31*; *RsMYB308L*, *RsMYB330L1* and *RsMYB330L2*, homologs of *AtMYB4*; [3, 15, 21, 26, 32, 46, 50, 79]).

## Discussion

In the present work, we have documented the effects of mutations in ABP genes–coding and regulatory–and the effects of expression of these genes on flower color variation. Our results demonstrate the importance of both structural and regulatory changes during ABP evolution in *Ruellia*. We identified 12 candidate ABP structural genes plus 9 candidate transcriptional regulators, including 4 MYBs, 2 WDRs, and 3 bHLHs among the 10 investigated species. Through co-expression network analyses, we found evidence for strong, correlated expression among most ABP structural genes as well as between most of these genes and one candidate regulatory MYB.

Phylogenetic relationships among the 10 species here investigated yield a hypothesis of four potential flower color transitions within the group: purple to red, red to yellow, red to purple, and purple to yellow (Fig. 1A). These four flower color transitions are consistent with patterns documented in a study with much more extensive phylogenetic sampling [74] and implicate both gain and loss of function mutations during flower color evolution in *Ruellia*. Prior studies on ABP have yielded evidence that four classes of mutations contribute importantly to color shifts across angiosperms: (1) mutations in ABP structural genes that cause either loss of function or changes to enzyme binding affinity, (2) mutations in cis-regulatory regions of ABP structural genes, and (3) mutations in coding regions or (4) cis-regions of ABP regulatory genes that impact expression of structural genes [17, 26, 37, 43, 49, 61, 63, 84, 88, 90, 92]. The purple to red color transition that occurred between the *R. breedlovei* lineage and the clade containing *R. fulgida* likely involved a loss of function mutation in the *F3′5'H* gene in the latter: our data showed that all essential ABP structural genes including *F3′5'H* itself were expressed at detectable levels (Fig. 2), but sequence analysis of *F3′5'H* revealed a premature stop codon caused by a C to T point mutation at positon 373 in *R. fulgida* (Additional file 8: Fig. S8). By contrast, both our de novo and reference-based approaches failed to assemble *F3′5'H* in the red-flowered species *R. brevifolia*, which belongs to the same clade as *R. fulgida*, and expression analysis indicated *F3′5'H* was expressed at extremely low levels (Fig. 2). These data indicate that a regulatory mutation was likely involved in this purple to red color transition. In the yellow-flowered species *R. bourgaei, R. speciosa*, and *R. lutea,* we observed down-regulation of multiple structural genes, including down-regulation of *F3H* in the closely related *R. bourgaei* and *R. speciosa* as well as down-regulation of *ANS* in all three species (Fig. 2). These results suggest either independent loss of functions in the *cis*-regulatory elements of these structural genes in the two clades or, more likely, mutations in shared regulatory genes given that expression of *F3H* and *ANS* is strongly coordinately regulated (Fig. 4). Finally, the shift from red to purple flowers that occurred between *Ruellia elegans* and *R. hirsuto-glandulosa* likely involved reactivation of the *F3′5'H* pathway branch. Although restoration of pathway function is expected to be more difficult than degradation of function [54, 66], putative gains in floral anthocyanins have been documented ([4], 31). In *R. elegans*, malvidin–a derivative of delphinidin–was detected in leaf tissues albeit in low concentrations, indicating functionality of the *F3′5'H* pathway in this species (Additional file 16: Data S2). Restoration of the *F3′5'H* pathway in petals of *R. hirsuto-glandulosa* may have been achieved by mutation in the *cis*-regulatory region of this enzyme, leading to decreased binding affinity of transcription inhibitors or increased binding affinity of transcription activators.

With exception of a few mutations in *F3'H2* and *DFR3* (Fig. 6) and two mutations yielding premature stop codons in *F3′5'H* (Additional file 8: Fig. S8), sequence comparisons and protein predictions of ABP structural genes among species with different flower colors indicated that the majority of amino acid mutations likely had neutral or minimally different functional effects, thus unlikely to result in enzyme dysfunction (Additional file 17: Data S3). Thus, much of the observed variation in flower colors and anthocyanin accumulation in *Ruellia* is more likely to be the result of mutations impacting the expression of ABP structural genes (Fig. 2). Regulatory mutations are considered to be among the most important factors in driving morphological evolution [7, 84] given that such changes can potentially lead to modification in expression of entire pathways. Through co-expression analysis, we found that expression of one of the R2R3 MYBs identified in our study–*MYB10L1*, which is homologous to the known ABP regulator *MdMYB10* in apples (*Malus* × *domestica*; 79)–was highly associated with several structural genes including *CHS, F3H*, *DFR1*, *DFR2*, and *ANS* (Fig. 4). Thus, mutations that affect *MYB10L1*, be they regulatory or coding, may potentially impact all five of these candidate structural genes. However, sequence comparison of *MYB10L1* across all species of *Ruellia* sampled here recovered no lethal mutations (Fig. 5, Additional file 17: Data S3), indicating *MYB10L1* itself is likely regulated by a higher order of regulators.

Notably, based on patterns of expression correlation with *MYB10L1*, the regulation of candidate ABP structural genes in our dataset can be separated into two distinct regulatory blocks (Fig. 4). Group 1 contains genes whose expression are likely under the control of *MYB10L1*: *CHS*, *F3H*, *F3′5'H* (significantly correlated with *DFR2,* r = 0.65*,* weakly associated with MYB10L1, r = 0.56), *DFR1*, *DFR2*, and *ANS.* Group 2 contains genes whose expression is independent of *MYB10L1*, among which *CHI* and *DFR3* seem to be regulated in a coordinated manner (Fig. 4). In contrast to these two blocks, each copy of *F3'H* and the single copy *UF3GT* are regulated independently. While independent regulation of *F3'H* has been observed in other studies, co-expression of *UF3GT* with *CHS* or *F3H* in *Arabidopsis* has been reported in the past [1, 63]. Thus, the observed expression pattern of *UF3GT* in the present study seems to be a unique feature of our dataset.

Prior research has demonstrated lower rates of non-synonymous mutations among genes upstream in the ABP pathway compared to downstream genes [53], likely as a function of relaxed evolutionary constraint on downstream (vs. upstream) genes [40] or higher rates of positive selection on downstream genes, potentially playing a role in adaptive floral color evolution [28]. This pattern is expected given that upstream enzymes function in a much broader set of biosynthetic pathways (e.g., lignin and other flavonoids) than downstream enzymes, which are largely specific to anthocyanin production (Additional file 9: Fig. S9). Data from *Ruellia* reveal similar patterns of rate evolution of ABP genes compared to prior studies. Specifically, two of the lowest dn/ds substitution rates were documented for *CHS* and *CHI* (Table 2), consistent with a hypothesis that these two genes are undergoing stronger purifying selection. Furthermore, across all candidate ABP loci, loss of expression was observed only in instances of genes downstream from *CHS* and *CHI* (Fig. 2), likely reflecting the essential role of these two enzymes in other metabolic pathways besides the ABP [86].

Flower color often reflects adaptation to pollinators [11]. For example, floral anthocyanin content has a direct effect on pollinator behavior [60] and plant fitness [11]. That all candidate structural genes identified in the present study are under purifying selection adds to evidence that selection acts to maintain overall primary protein function of ABP genes. However, our data also yielded evidence that selection may drive diversification of ABP products in some lineages, specifically, we found signatures of positive selection in several sites in *DFR* and *F3H* across species (Fig. 6), and some of these sites are located in or nearby protein binding regions (PBR). This is especially the case for *DFR3*, in which five out of seven sites under positive selection are located within predicted PBR Mutations occurring at these sites are more likely to impact substrate binding affinities of the corresponding enzyme resulting in new interactions [55]. In this study, we found multiple, orthologous copies of both *F3H* and *DFR* to be present and here hypothesize that duplication of these genes may have facilitated functional diversification of the ABP in *Ruellia* [28].

Transcription factors (TFs) are key players in regulating flux through secondary metabolic pathways by controlling relative levels of gene expression [13]. To further investigate regulation of the ABP in the context of potential interactions with other metabolic pathways, we conducted a phylogenetic study of candidate MYBs as well as co-expression analyses of broader diversity of TFs across all species. We identified nine candidate R2R3 MYBs associated with ABP genes in *Ruellia*. Among these, orthologs of *RsMYB10L1*, *RsMYB114L* (i.e., *AtMYB75*, [10]), *RsMYB305L1*, and *RsMYB305L2* (i.e., *NtMYB305*; Wang et al. [79]) have been reported to function in regulating anthocyanin accumulation. Our investigation demonstrated that *RsMYB10L1* expression is highly correlated with five ABP structural genes (Fig. 4), and phylogenetic analyses demonstrated that *RsMYB10L1* belongs to a strongly supported clade that contains *AtMYB75* orthologs (Additional file 7: Fig. S7). This result in combination with sequence analysis and other expression patterns (Fig. 5) indicate specifically that *RsMYB10L1* is a very strong candidate regulator of the ABP in *Ruellia*. Pilot experimentation with virus-induced gene silencing focusing on ABP knockdowns of *RsMYB10L1* yielded additional support for this candidate (Zhuang and Manzitto-Tripp, unpub. data; Additional file 10: Fig. S10). Our phylogenetic analyses placed the remaining *Ruellia* R2R3 MYBs in a clade with *Arabidopsis* orthologs that function in lignin biosynthesis (e.g., *AtMYB4, AtMYB31*, and *AtMYB46/AtMYB83*, Additional file 7: Fig. S7; [3, 15, 32, 46, 50]. These results support a hypothesis of different functional roles of the R2R3 MYBs recovered in *Ruellia–*some in ABP production vs. others potentially in lignin production–albeit in pathways that interact via common upstream enzymes (Additional file 9: Fig. S9, Tohge et al. [73]). Functionally, anthocyanins and lignins are both proposed to protect plants against UV radiation [44], and redirection of metabolic fluxes between the lignin and flavonoid pathways has been observed in many plant species [57]. Over-expression of *AmMYB308* in transgenic tobacco plants and several of its orthologs belonging to the same clade led to significant repression of lignin biosynthesis and increased total flavonoids [42, 71]. Other studies have similarly demonstrated MYBs as negative regulators of lignin content [3] whereas others likely serve as activators of lignin biosynthesis [12, 32, 96]. Taken together, data suggest extensive interactions between anthocyanin and lignin biosynthesis through modification of expression of relevant TFs. As such, future attempts to understand regulatory impacts on flower color evolution in *Ruellia* would optimally incorporate information from other biosynthetic pathways that likely interact with anthocyanin production (e.g., [76]).

We also identified several TFs that are highly co-expressed with ABP-associated TFs in *Ruellia*. These include auxin responsive factors (ARFs), abscisic acid signaling genes (ABA), and other TFs, some of which function as activators and others as repressors based on prior studies in other plants (Additional file 5: Fig. S5; [18, 25, 48, 77, 83]). In the present study, we found positive co-expression between two auxin signaling pathway activators *ARF6* and *ARF19* [48, 77] and ABP-associated TFs in *Ruellia*. We speculate that this positive association results from the role of auxin in flower development. Specifically, the accumulation of anthocyanins gradually increases during flower development in *Ruellia* (shown in Fig. 3A). Auxin is required for the initiation of floral primordia and disruption of auxin biosynthesis leads to the failure of flower formation [16]. Our hypothesis is further supported by the observed correlation of a group of MADS-box TFs with ABP structural genes, which is exclusively expressed only in petal tissues (Additional file 11: Fig. S11). Sequence analysis indicated one gene was homologous to *AGL6*, two genes were homologous to *MADS1*, and another two genes were homologous to *MADS2*, all of which impact flower development [41, 61]. Taken together, our data reveal strong, coordinated regulation between anthocyanin accumulation and flower development.

## Conclusions

The ABP is one of the most well-studied secondary metabolic pathways in plants. Although key structural genes have been extensively characterized and are highly conserved across unrelated species [52], the complex mechanisms regulating the ABP are still not fully understood largely as a result of prior emphasis on model plant systems. The present study lays the first steps towards understanding molecular evolution and expression of ABP genes in *Ruellia*. Specifically, it yields a first comprehensive survey of candidate structural and regulatory loci involved in the production of diverse flower colors in the genus. Data from our expression and co-expression analyses indicate that regulatory mutations have likely been more important in color shifts but that mutations to structural genes have also played a role. Finally, different portions of the ABP appear to be under coordinated regulation such that a single 'regulatory block' is unlikely to characterize the entire pathway, perhaps as a function of extensive interactions with other metabolic pathways.

## Supplementary Information

**Additional file 1.** **Figure S1.** Flowchart summarizing major steps in our data analysis protocol.

**Additional file 2.** **Figure S2.** MAFFT-aligned amino acid sequences of 12 assembled candidate ABP structural genes.

**Additional file 3.** **Figure S3.** **A**. Results from phylogenetic analysis of candidate anthocyanin WD40 regulators identified in Ruellia and their orthologs in other species. **B**. Protein sequence alignment of candidate anthocyanin WD40 regulators identified in Ruellia and their orthologs in other species. Relative expression of each gene shown as Fragments Per Kilobase of transcript per Million mapped reads (FPKM).

**Additional file 4.** **Figure S4. A**. Results from phylogenetic analysis of candidate anthocyanin bHLH regulators identified in Ruellia and their orthologs in other species. **B**. Protein sequence alignment of candidate anthocyanin bHLH regulators identified in Ruellia and their orthologs in other species. Relative expression of each gene shown as Fragments Per Kilobase of transcript per Million mapped reads (FPKM).

**Additional file 5.** **Figure S5. A**. Functional classification of KEGG pathway transcripts identified in Ruellia and found to be significantly co-expressed with Ruellia ABP structural genes. The KEGG pathways were summarized into four main categories: Cellular Processes, Environmental Information Processing, Genetic Information Processing, and Metabolism. **B**. Pie chart displaying the distribution of ABP-associated transcription factors recovered among transcripts of Ruellia.

**Additional file 6.** **Figure S6.** ABP-associated KEGG pathways identified by co-expression analysis. All bottom-level categories were shown. Total number of transcripts for each category was shown in y axis.

**Additional file 7.** **Figure S7.** Phylogenetic tree showing relationships among 12 ABP-associated R2R3-MYB genes identified from Ruellia simplex and 132 MYB genes from Arabidopsis thaliana. The tree was constructed using the PROTGAMMAWAG model implemented in RAxML version 8 (Stamatakis, 2014). The 12 R2R3-MYB genes from Rullia simplex are highlighted in red.

**Additional file 8.** **Figure S8.** cDNA sequence alignment of assembled F3'5'H genes from seven Ruellia species. Red arrows showing mutation sites that introduce premature stop codons in Ruellia lutea (bp: 145) and Ruellia fulgida (bp: 367), respectively.

**Additional file 9.** **Figure S9.** Simplified phenylpropanoid pathway for the biosynthesis of anthocyanins and lignins.

**Additional file 10.** **Figure S10.** Functional validation of RsMYB10L in R. simplex. A. R. simplex flower without virus infection. B. R. simplex flower infected by virus carrying 247bp cDNA fragment cloned from RsMYB10L

**Additional file 11.** **Figure S11.** Heat map of ABP-associated transcription factors (TFs). Green represents genes with relatively high expression levels and red represents genes with relatively low expression levels. For each TF group shown to the far right, genes were ordered based on their overall expression levels in Ruellia brevifolia. MADS-box type TF shown with a red star.

**Additional file 12: Table S1.** Results from attempted assembly of structural genes in the ABP in Ruellia. Genes that were assembled in either the leaf (L) or petal (P) tissue shown. Genes that failed to be assembled are shown via an "-". Genes terminated by a premature stop codon are shown via X. The presence of pelargonidins, cyanidins, and/or delphinidins shown via the first three columns.

**Additional file 13: Table S2.** Custom primers used in the qRT-PCR analysis.

**Additional file 14: Table S3.** Pearson correlation coefficents (r) among identified R2R3 MYBs and ABP structural genes. Values of r ≥ 0.65 are shown in bold and the highest r value for each MYB are highlighted in red.

**Additional file 15.** **Table S1.** Results from attempted assembly of structural genes in the ABP in Ruellia. Genes that were assembled in either the leaf (L) or petal (P) tissue shown. Genes that failed to be assembled are shown via an "-". Genes terminated by a premature stop codon are shown via X. The presence of pelargonidins, cyanidins, and/or delphinidins shown via the first three columns.

**Additional file 16.** **Table S2.** Custom primers used in the qRT-PCR analysis.

**Additional file 17.** **Table S3.** Pearson correlation coefficents (r) among identified R2R3 MYBs and ABP structural genes. Values of r ≥ 0.65 are shown in bold and the highest r value for each MYB are highlighted in red.

## Data Availability

The datasets generated and analyzed during the present study are available at http://www.ncbi.nlm.nih.gov/bioproject/PRJNA323650.

## Abbreviations

Busco: Being near-universal single-copy genes
cDNA: Complementary DNA
CDS: Coding sequence
HCl: Hydrochloric acid
KEGG: Kyoto Encyclopedia of Genes and Genomes
ORF: Open reading frame
NCBI: National Center for Biotechnology Information
RNA: Ribonucleic acid
SEECER: Sequencing error correction for RNA reads
