# Supplementary figures and images for: Co-expression network analyses of anthocyanin biosynthesis genes in Ruellia (Wild Petunias; Acanthaceae)
Source: BMC Ecol Evol. 2022 Mar 8;22:27. doi: 10.1186/s12862-021-01955-x (PMC8905905; doi:10.1186/s12862-021-01955-x)

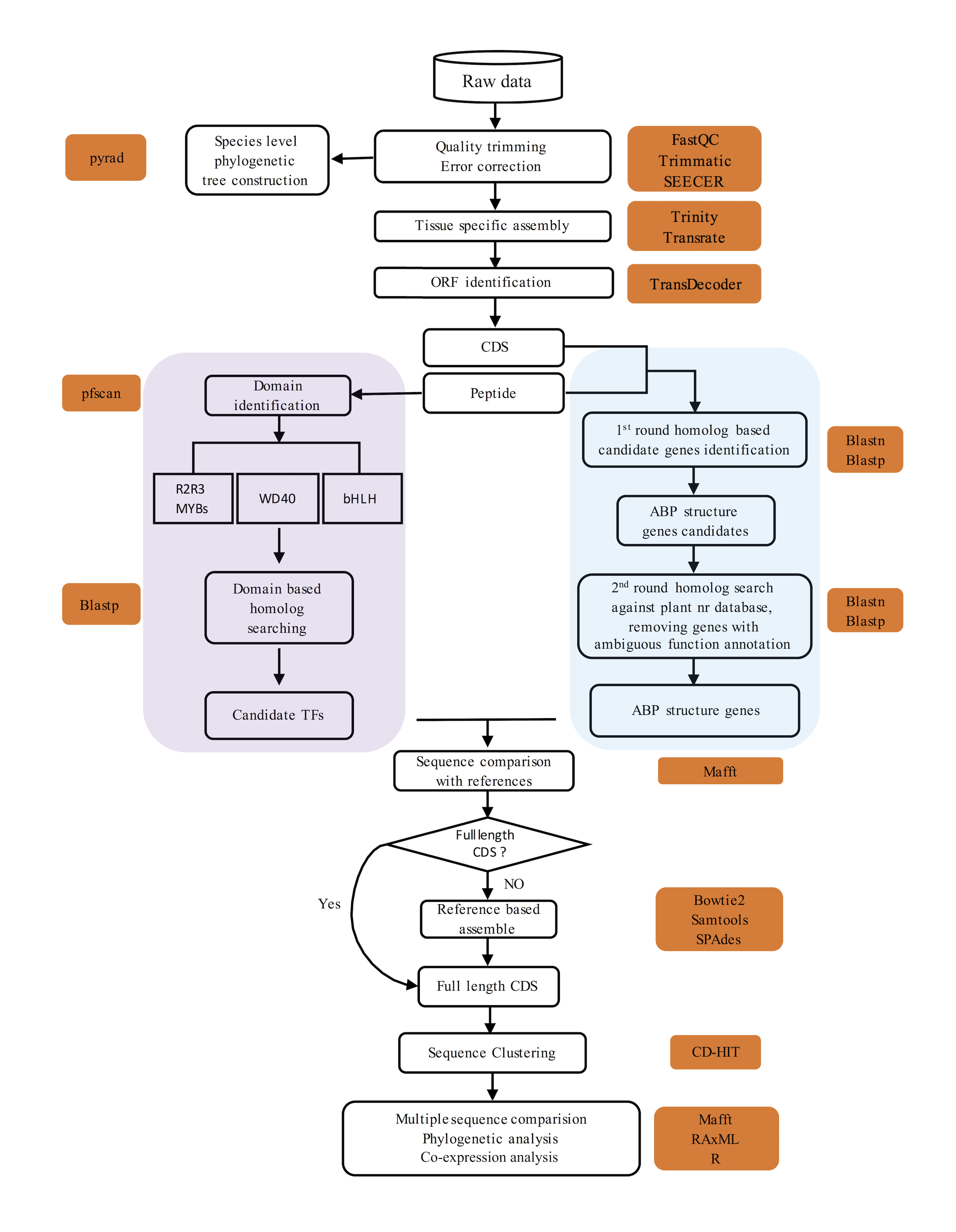

Supplement: Supplementary file 1 — Additional file 1. Figure S1. Flowchart summarizing major steps in our data analysis protocol. [file 12862_2021_1955_MOESM1_ESM.jpg]

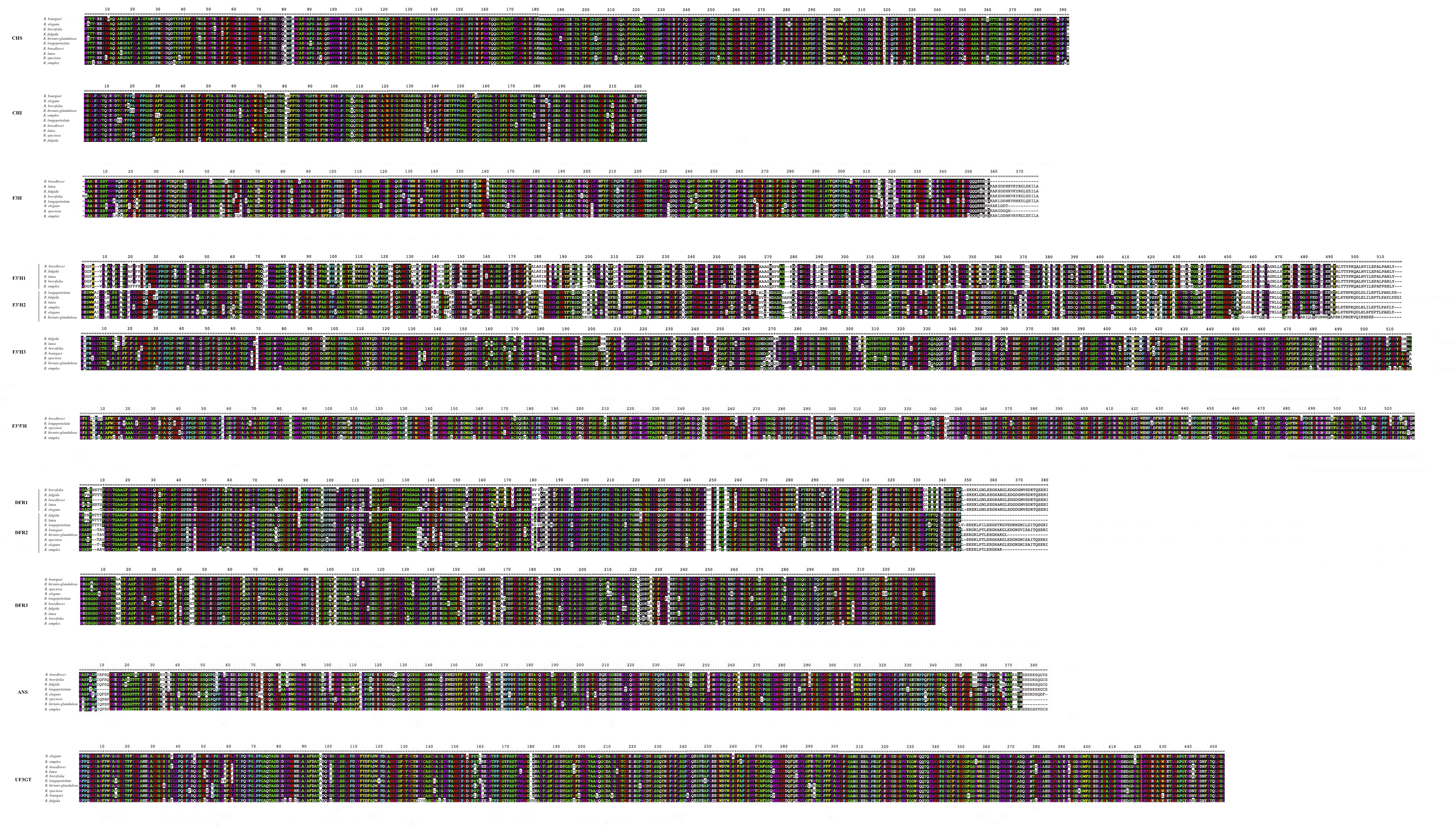

Supplement: Supplementary file 2 — Additional file 2. Figure S2. MAFFT-aligned amino acid sequences of 12 assembled candidate ABP structural genes. [file 12862_2021_1955_MOESM2_ESM.jpg]

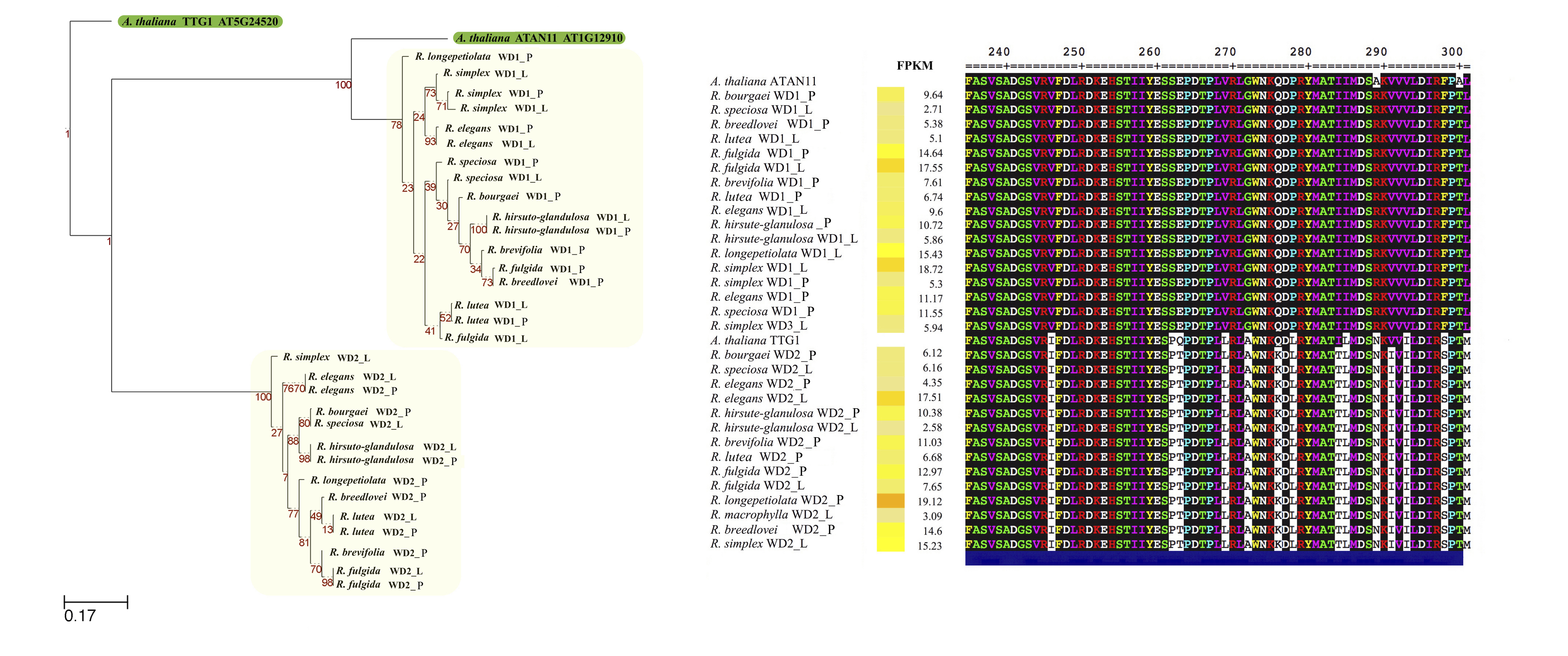

Supplement: Supplementary file 3 — Additional file 3. Figure S3. A. Results from phylogenetic analysis of candidate anthocyanin WD40 regulators identified in Ruellia and their orthologs in other species. B. Protein sequence alignment of candidate anthocyanin WD40 regulators identified in Ruellia and their orthologs in other species. Relative expression of each gene shown as Fragments Per Kilobase of transcript per Million mapped reads (FPKM). [file 12862_2021_1955_MOESM3_ESM.jpg]

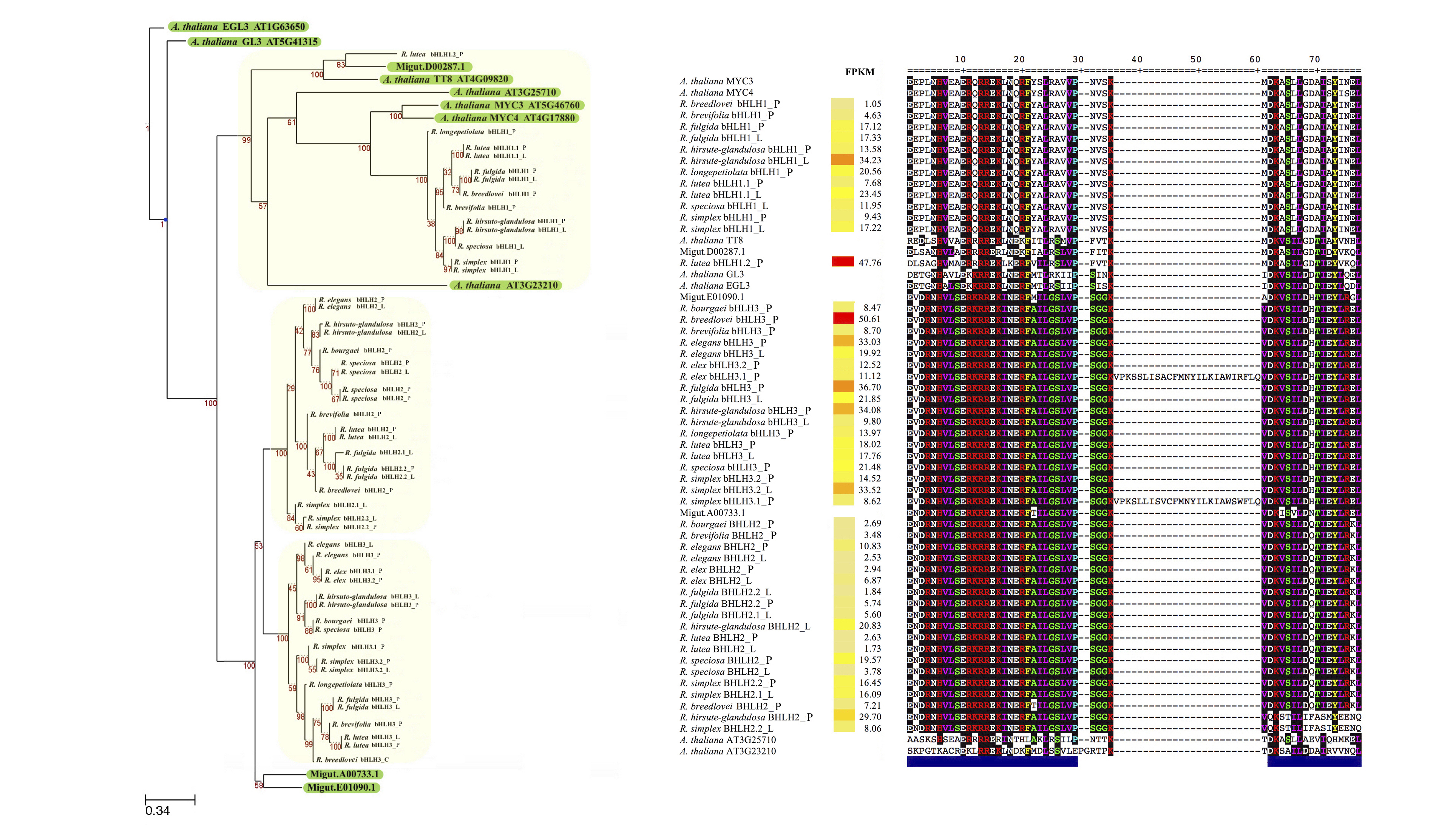

Supplement: Supplementary file 4 — Additional file 4. Figure S4. A. Results from phylogenetic analysis of candidate anthocyanin bHLH regulators identified in Ruellia and their orthologs in other species. B. Protein sequence alignment of candidate anthocyanin bHLH regulators identified in Ruellia and their orthologs in other species. Relative expression of each gene shown as Fragments Per Kilobase of transcript per Million mapped reads (FPKM). [file 12862_2021_1955_MOESM4_ESM.jpg]

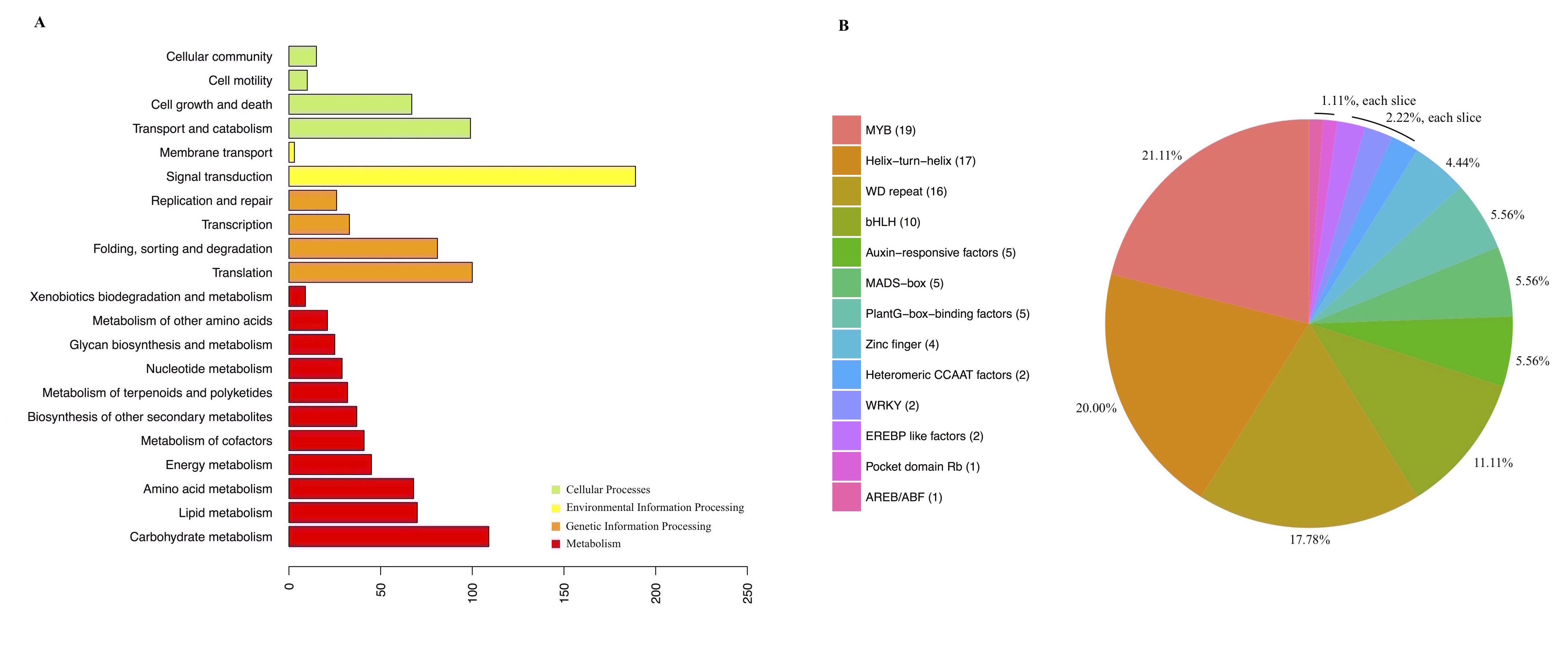

Supplement: Supplementary file 5 — Additional file 5. Figure S5. A. Functional classification of KEGG pathway transcripts identified in Ruellia and found to be significantly co-expressed with Ruellia ABP structural genes. The KEGG pathways were summarized into four main categories: Cellular Processes, Environmental Information Processing, Genetic Information Processing, and Metabolism. B. Pie chart displaying the distribution of ABP-associated transcription factors recovered among transcripts of Ruellia. [file 12862_2021_1955_MOESM5_ESM.jpg]

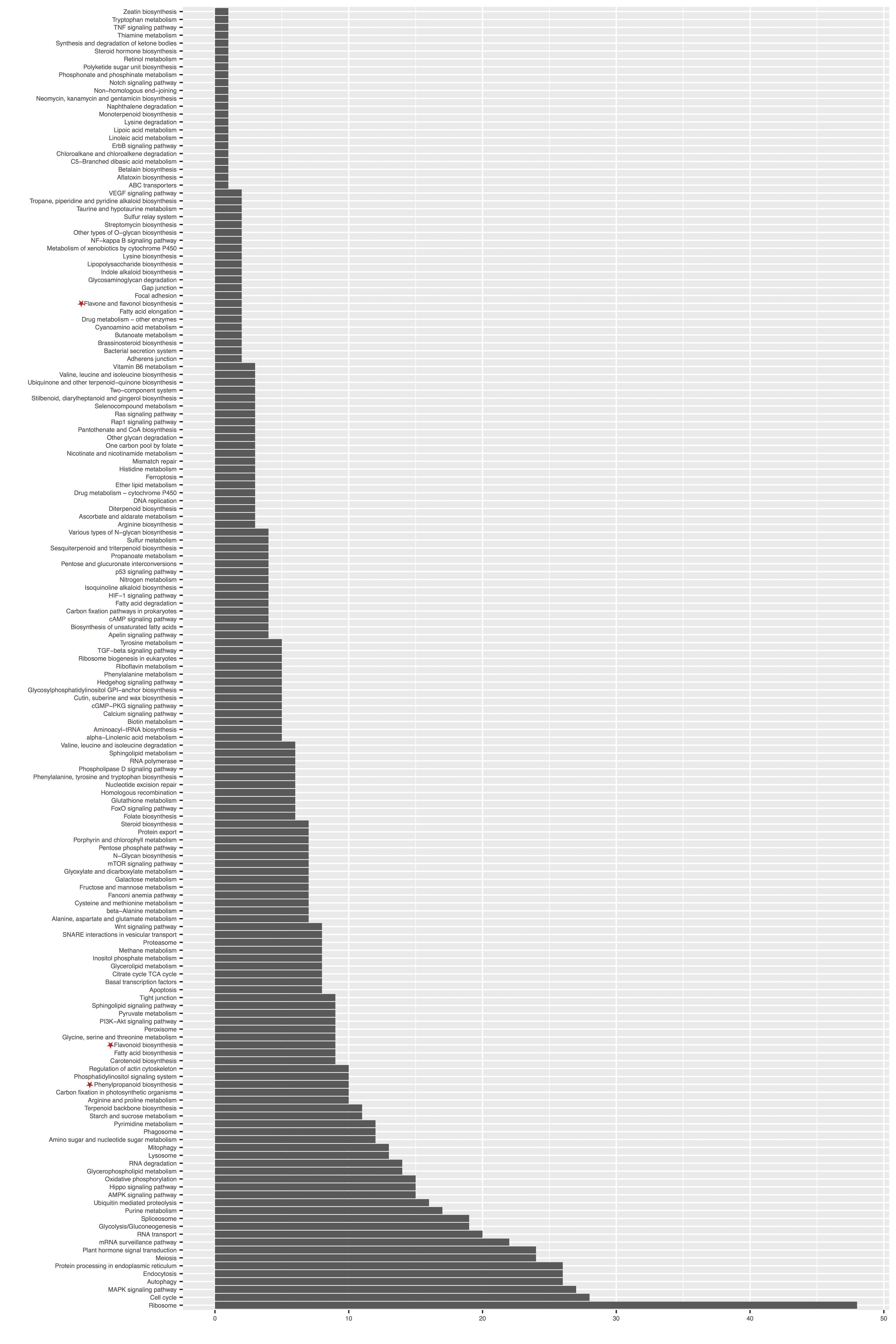

Supplement: Supplementary file 6 — Additional file 6. Figure S6. ABP-associated KEGG pathways identified by co-expression analysis. All bottom-level categories were shown. Total number of transcripts for each category was shown in y axis. [file 12862_2021_1955_MOESM6_ESM.jpg]

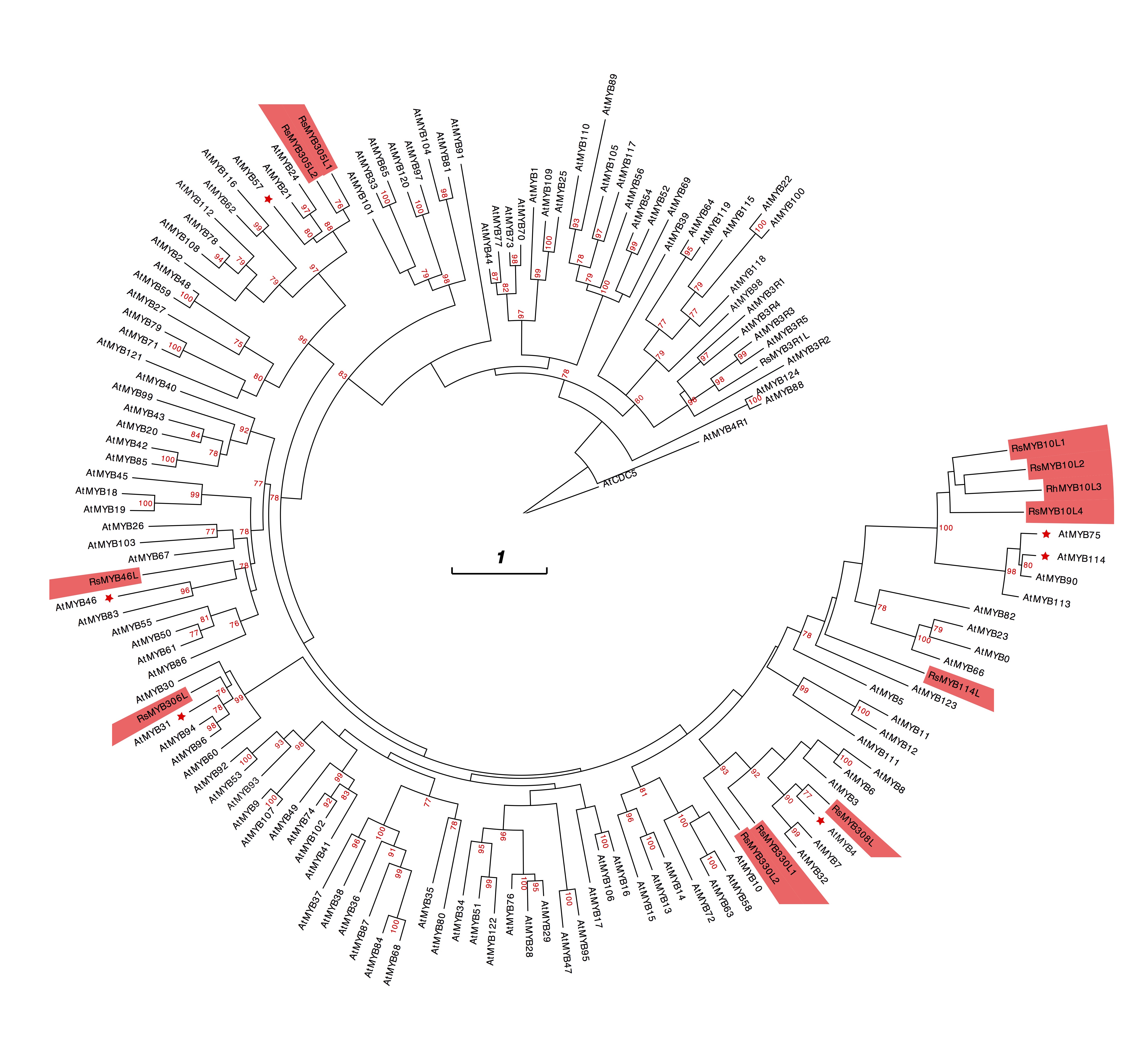

Supplement: Supplementary file 7 — Additional file 7. Figure S7. Phylogenetic tree showing relationships among 12 ABP-associated R2R3-MYB genes identified from Ruellia simplex and 132 MYB genes from Arabidopsis thaliana. The tree was constructed using the PROTGAMMAWAG model implemented in RAxML version 8 (Stamatakis, 2014). The 12 R2R3-MYB genes from Rullia simplex are highlighted in red. [file 12862_2021_1955_MOESM7_ESM.jpg]

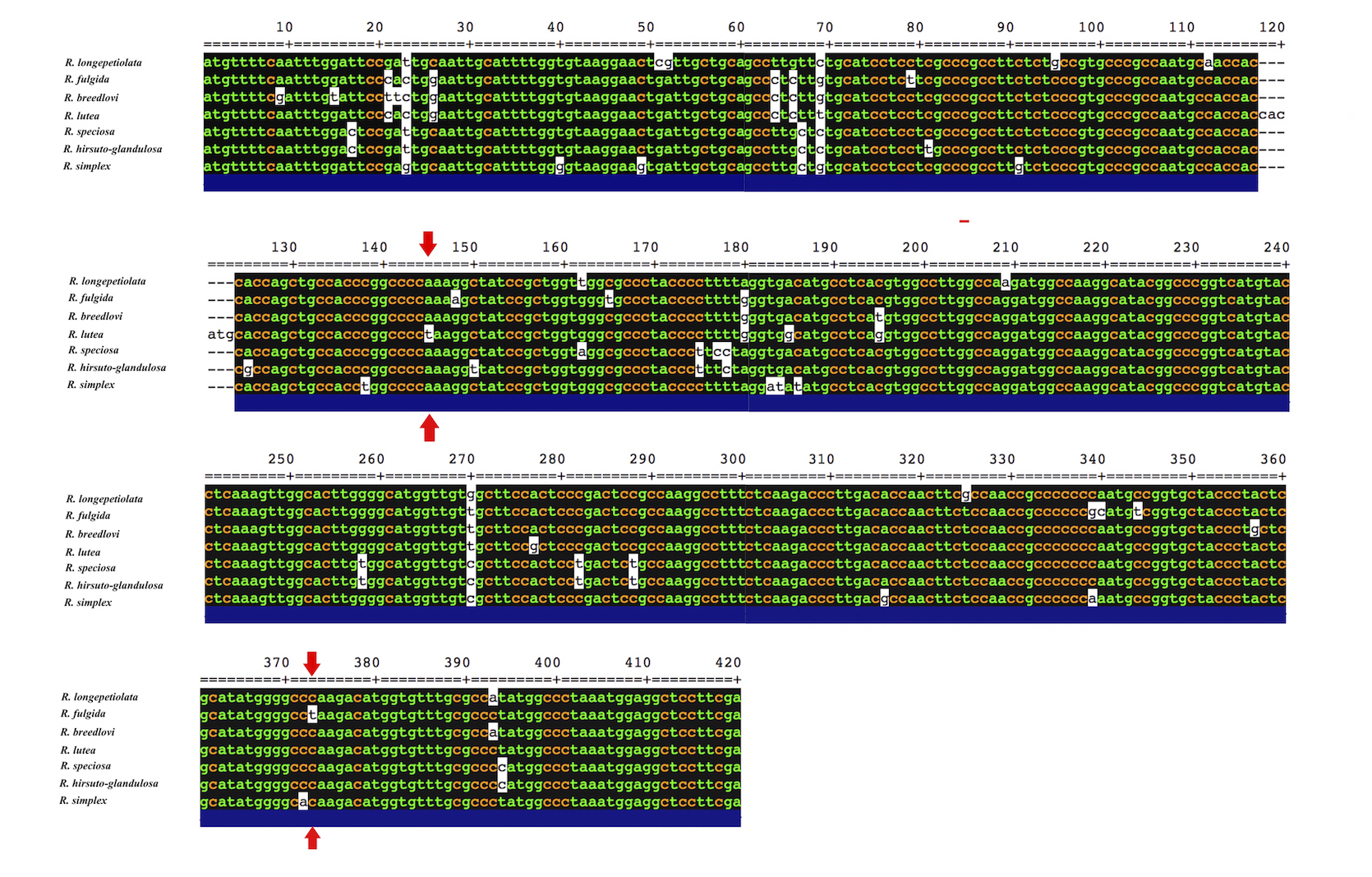

Supplement: Supplementary file 8 — Additional file 8. Figure S8. cDNA sequence alignment of assembled F3'5'H genes from seven Ruellia species. Red arrows showing mutation sites that introduce premature stop codons in Ruellia lutea (bp: 145) and Ruellia fulgida (bp: 367), respectively. [file 12862_2021_1955_MOESM8_ESM.jpg]

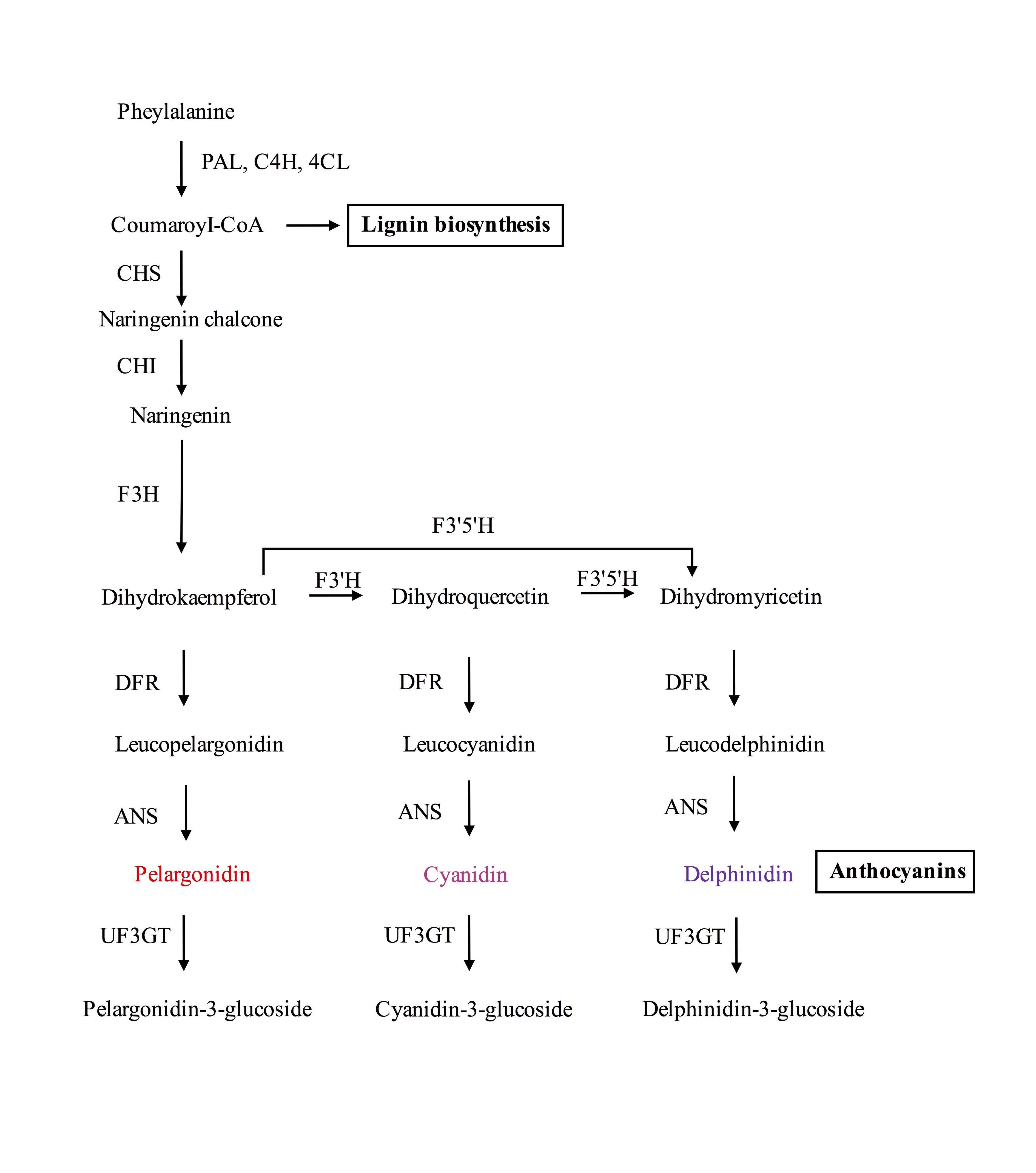

Supplement: Supplementary file 9 — Additional file 9. Figure S9. Simplified phenylpropanoid pathway for the biosynthesis of anthocyanins and lignins. [file 12862_2021_1955_MOESM9_ESM.jpg]

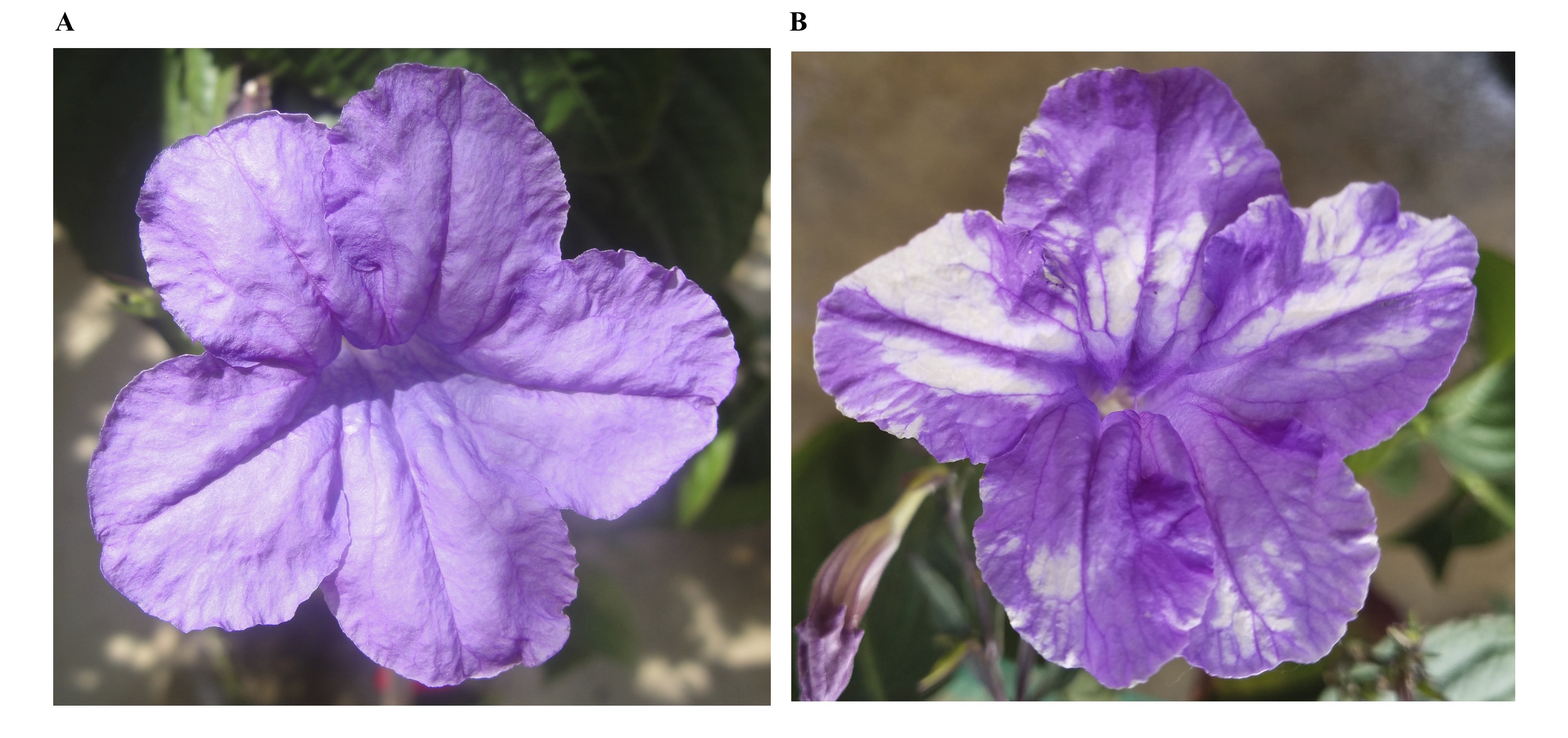

Supplement: Supplementary file 10 — Additional file 10. Figure S10. Functional validation of RsMYB10L in R. simplex. A. R. simplex flower without virus infection. B. R. simplex flower infected by virus carrying 247bp cDNA fragment cloned from RsMYB10L [file 12862_2021_1955_MOESM10_ESM.jpg]

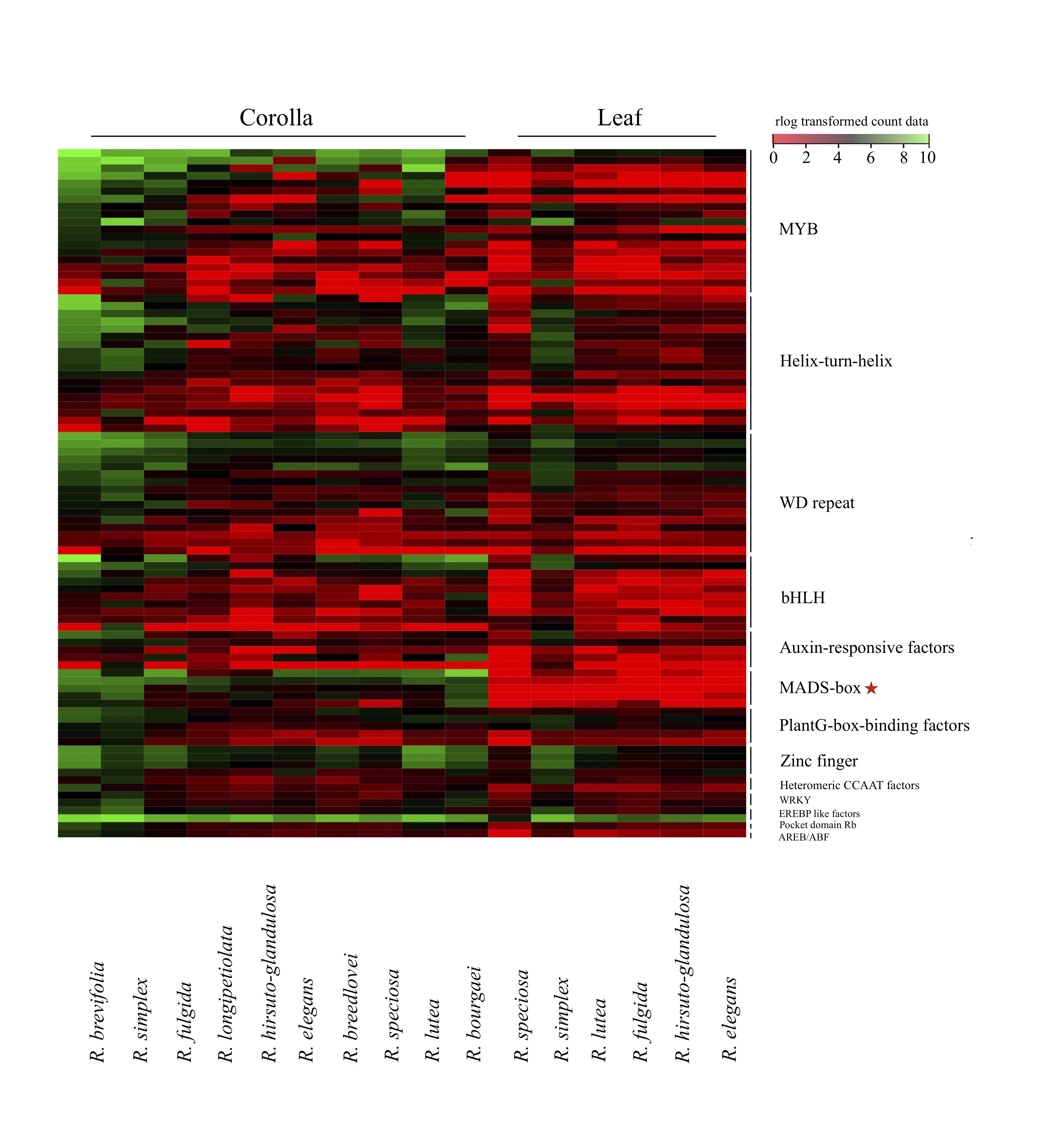

Supplement: Supplementary file 11 — Additional file 11. Figure S11. Heat map of ABP-associated transcription factors (TFs). Green represents genes with relatively high expression levels and red represents genes with relatively low expression levels. For each TF group shown to the far right, genes were ordered based on their overall expression levels in Ruellia brevifolia. MADS-box type TF shown with a red star. [file 12862_2021_1955_MOESM11_ESM.jpg]
