# Supplementary material for: Co-expression network analyses of anthocyanin biosynthesis genes in Ruellia (Wild Petunias; Acanthaceae)
Source: BMC Ecol Evol. 2022 Mar 8;22:27. doi: 10.1186/s12862-021-01955-x (PMC8905905; doi:10.1186/s12862-021-01955-x)
Supplement: Supplementary file 15 — Additional file 15. Table S1. Results from attempted assembly of structural genes in the ABP in Ruellia. Genes that were assembled in either the leaf (L) or petal (P) tissue shown. Genes that failed to be assembled are shown via an "-". Genes terminated by a premature stop codon are shown via X. The presence of pelargonidins, cyanidins, and/or delphinidins shown via the first three columns. [file 12862_2021_1955_MOESM15_ESM.docx]

**Data S1.** Floral reflectance spectra generated via spectrophotometry and used to assign flower colors to species (see main text). The median wavelength for each sample was used to bin into one of five flower colors as follows: ~600–650nm = red; ~420–460 *and* 600–650nm = pink; ~420–460nm = purple; ~500–550nm = yellow; ~420–700nm = white (no white species sampled in the present study). Different, usually overlapping spectral profiles represent two to three technical replicates measured on the same corolla. The wild voucher collection, representing the source of the floral reflectance traces, is provided. Delimited color is provided following each voucher name and number.

Ruellia bourgaei

Tripp & Kiel 428

Yellow

Ruellia breedlovei

Tripp et al. 4590

Purple

Ruellia brevifolia

Tripp et al. 5970

Red

Ruellia elegans

Tripp et al. 4594

Red

Ruellia fulgida

Tripp & Luján 494

Red

Ruellia hirsuto-glandulosa

Tripp et al. 5904

Purple

Ruellia longipetiolata

Tripp 311

Purple

Ruellia lutea

Tripp et al. 4598

Yellow

Ruellia simplex

Cultivated, COLO Greenhouses

Purple

Ruellia speciosa

Tripp & Acosta 175

Yellow
