# Supplementary material for: Co-expression network analyses of anthocyanin biosynthesis genes in Ruellia (Wild Petunias; Acanthaceae)
Source: BMC Ecol Evol. 2022 Mar 8;22:27. doi: 10.1186/s12862-021-01955-x (PMC8905905; doi:10.1186/s12862-021-01955-x)

R. bourgaei petal

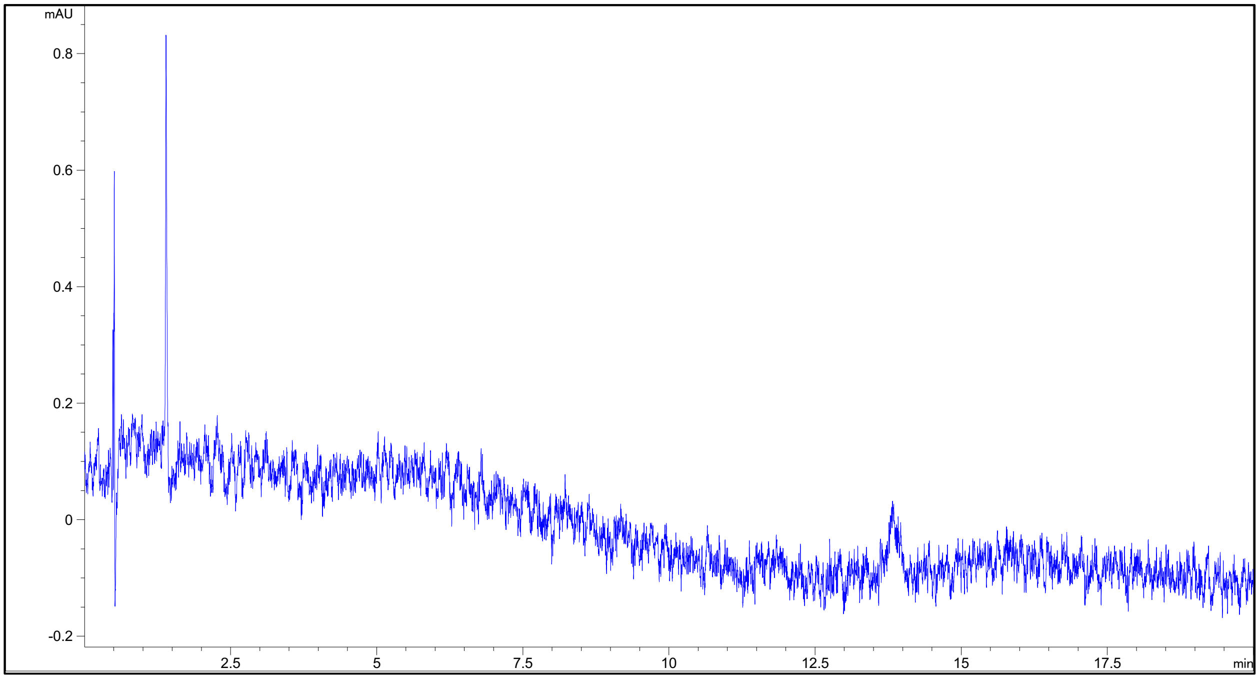

minutes

R. breedlovi petal

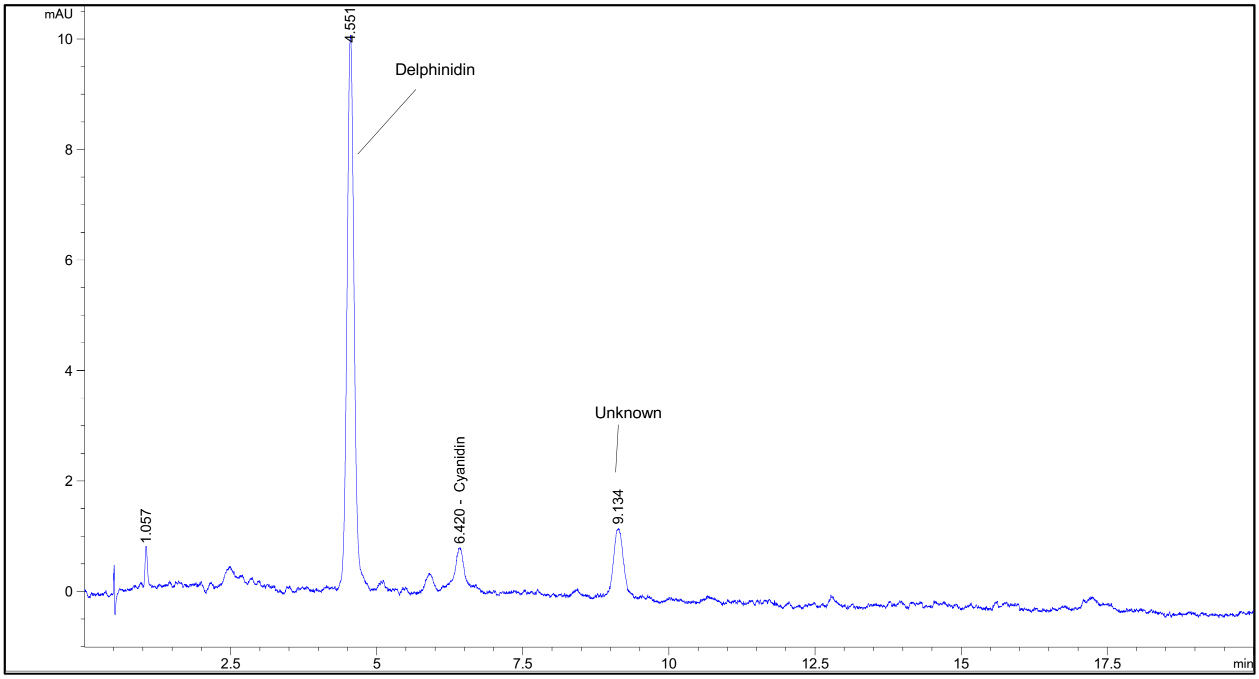

minutes

R. brevifolia petal

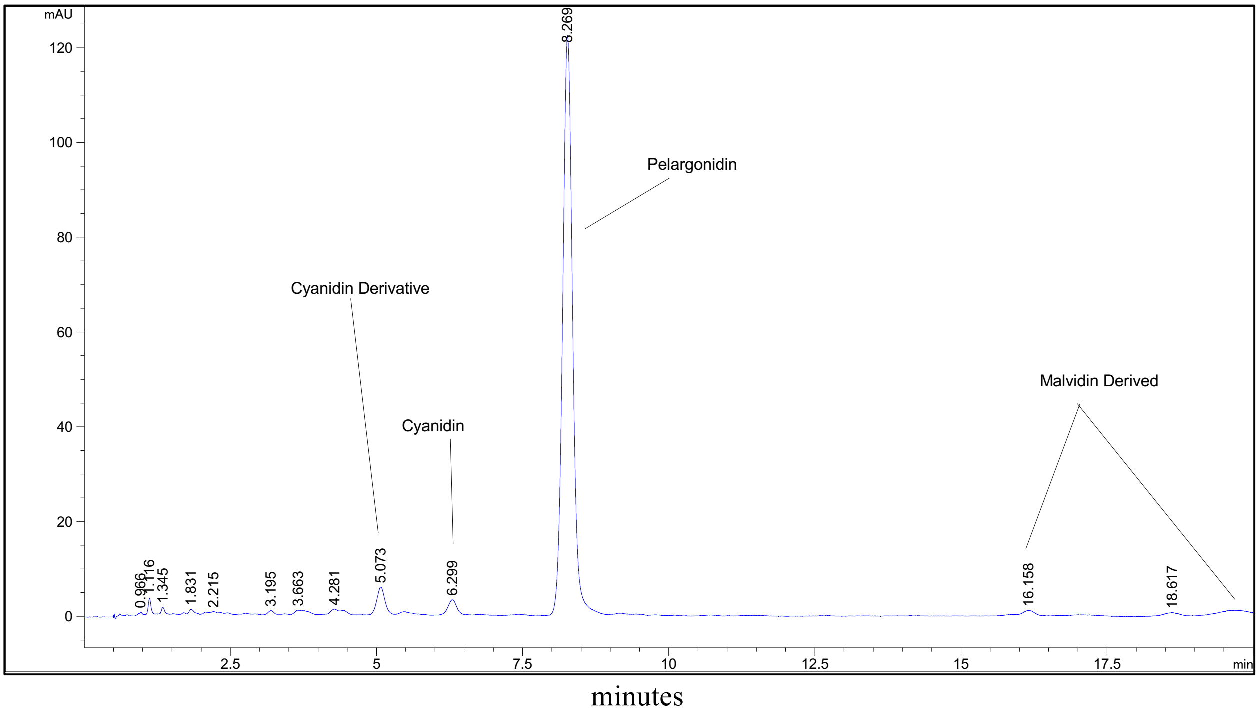

R. fulgida petal

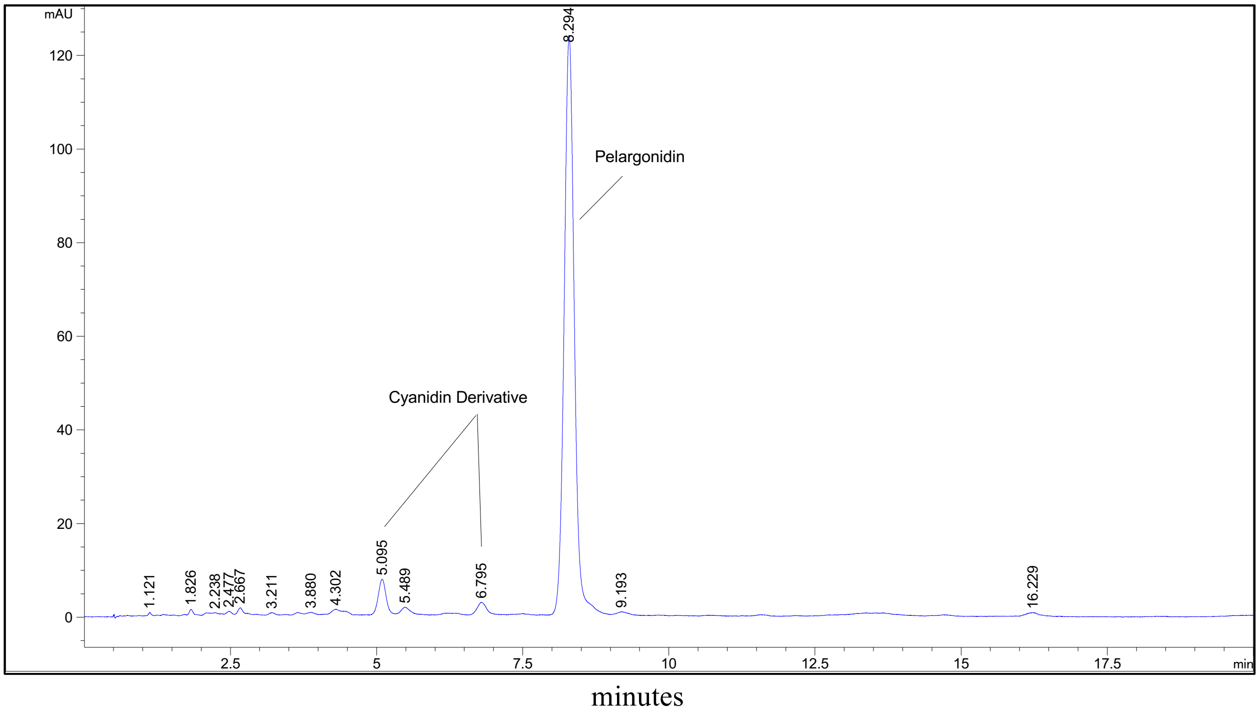

R. hirsuto-glandulosa petal

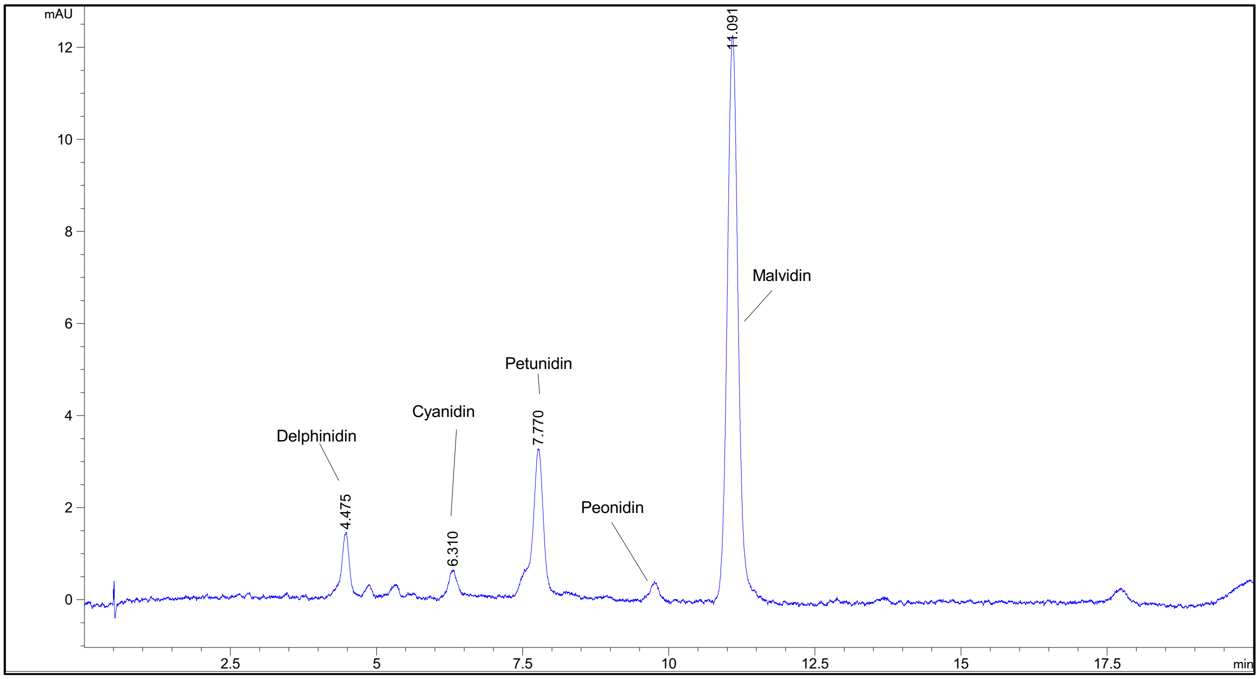

minutes

R. longipetiolata petal

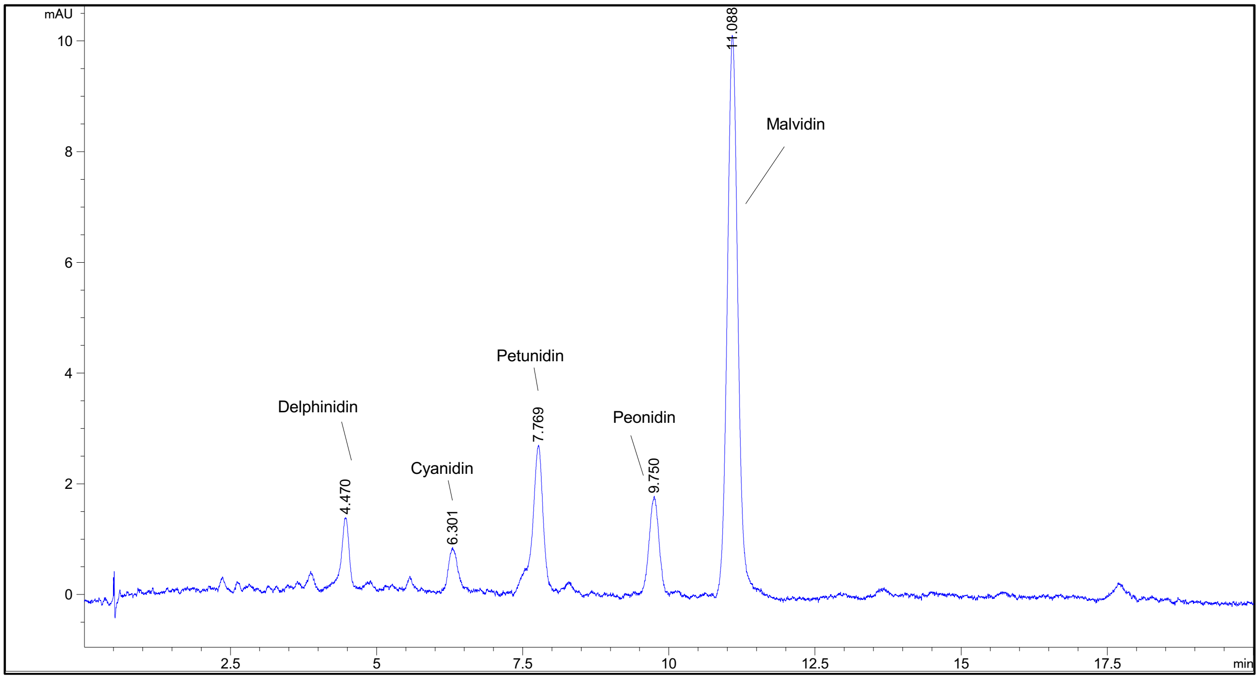

minutes

R. elegans petal

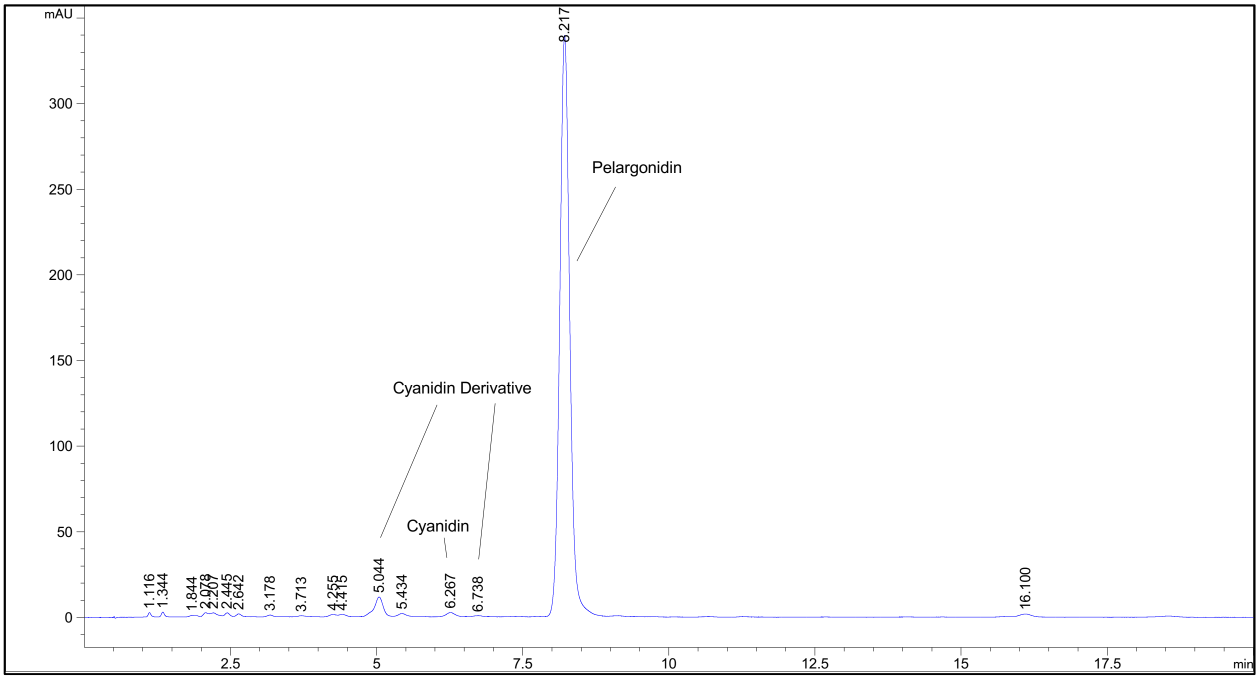

minutes

R. simplex petal

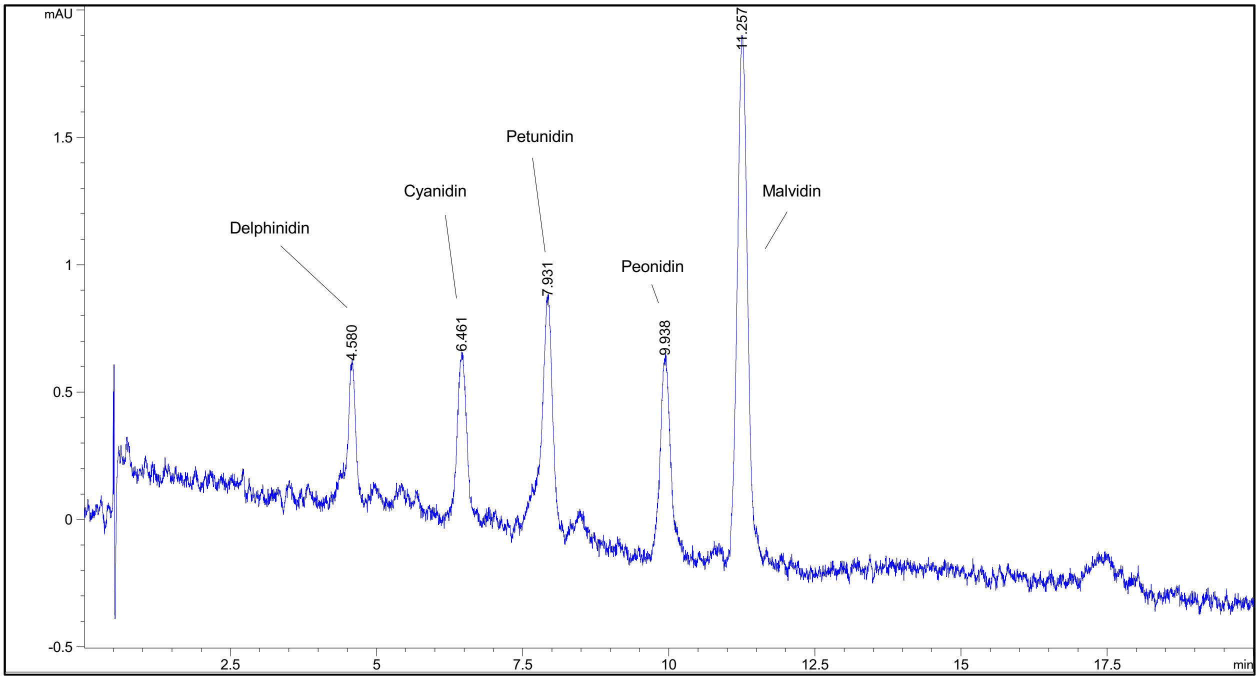

minutes

R. lutea petal

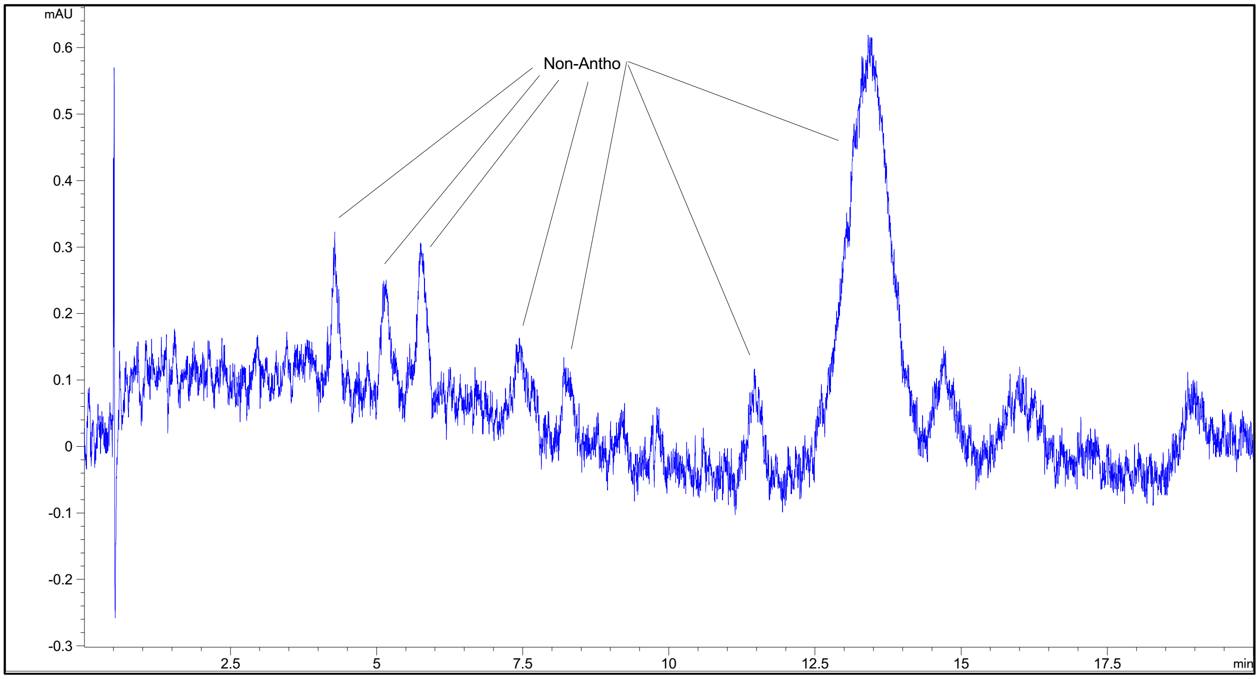

minutes

R. elegans leaf

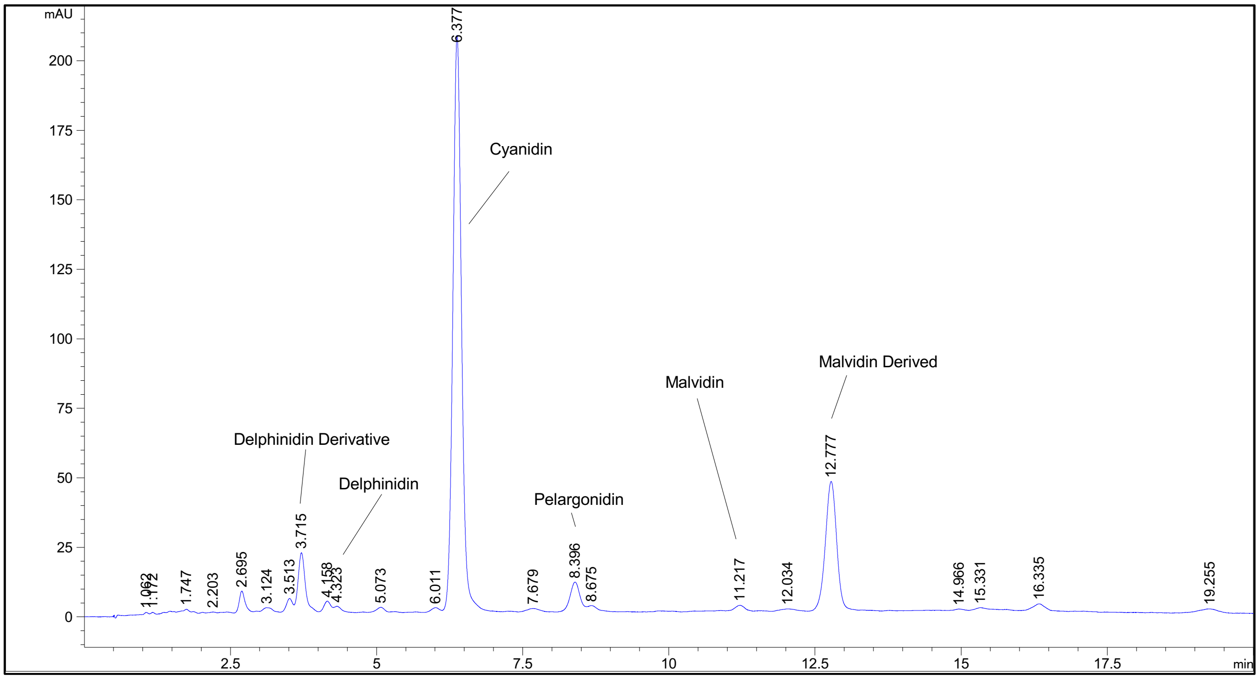

minutes

R. longipetiolata leaf

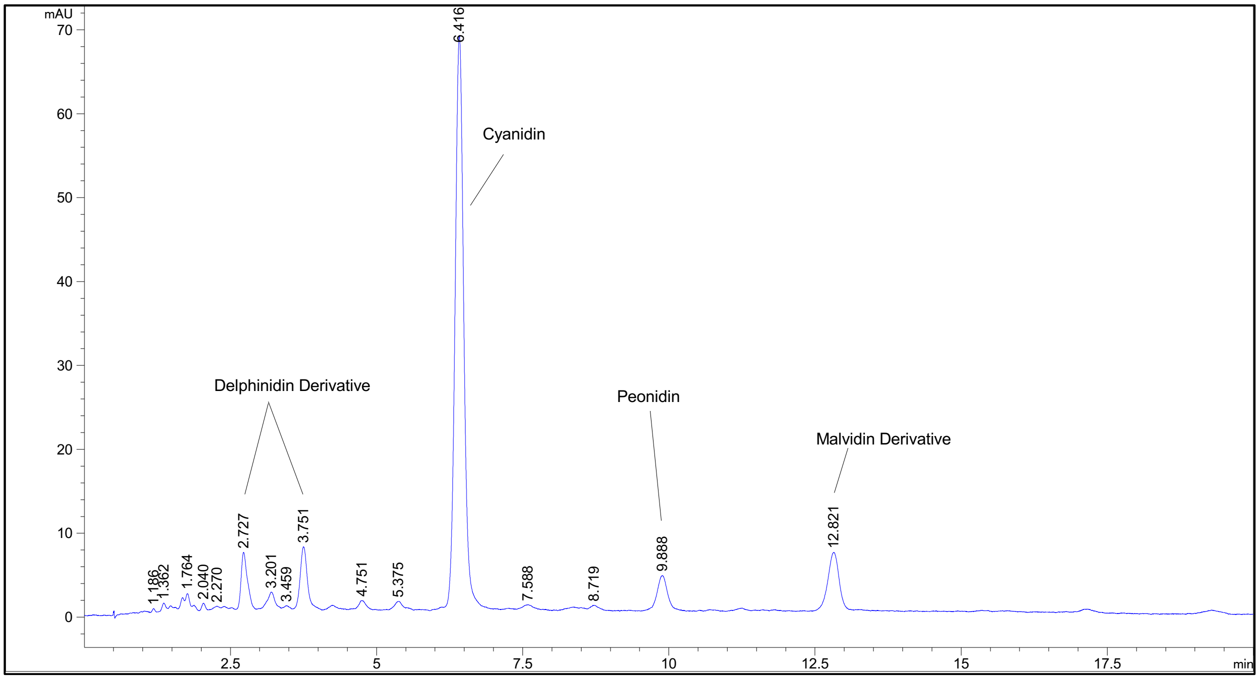

minutes

R. breedlovei leaf

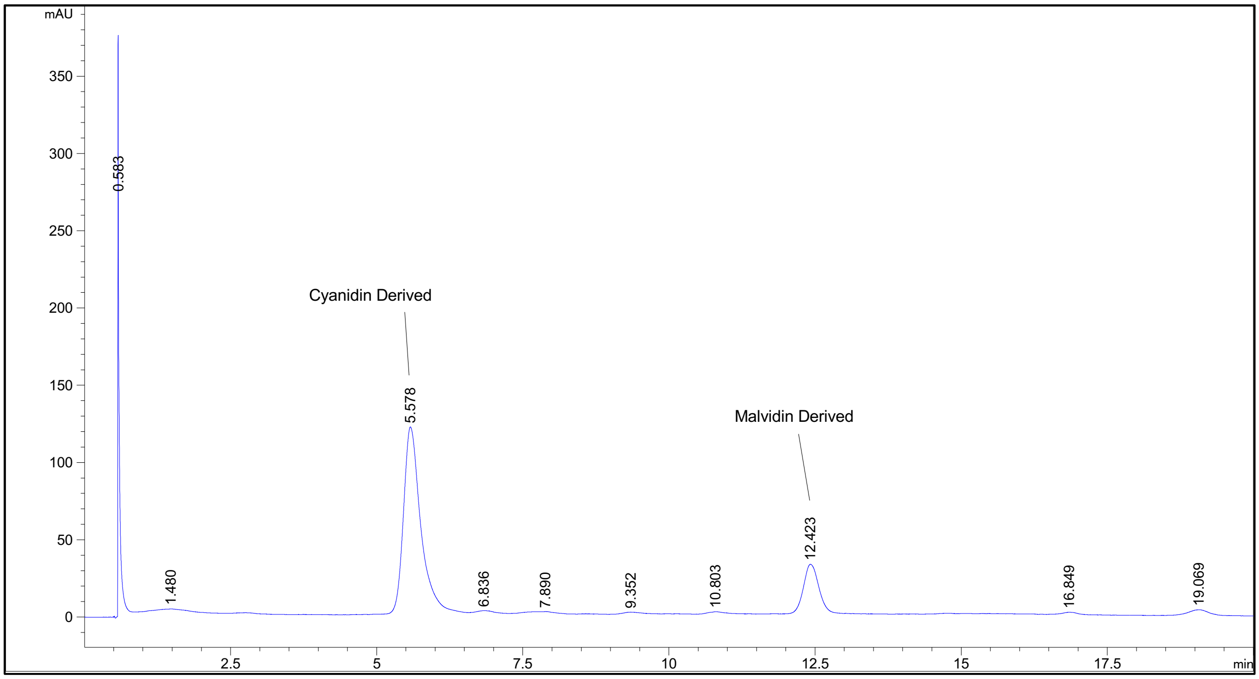

minutes

R. bourgaei leaf

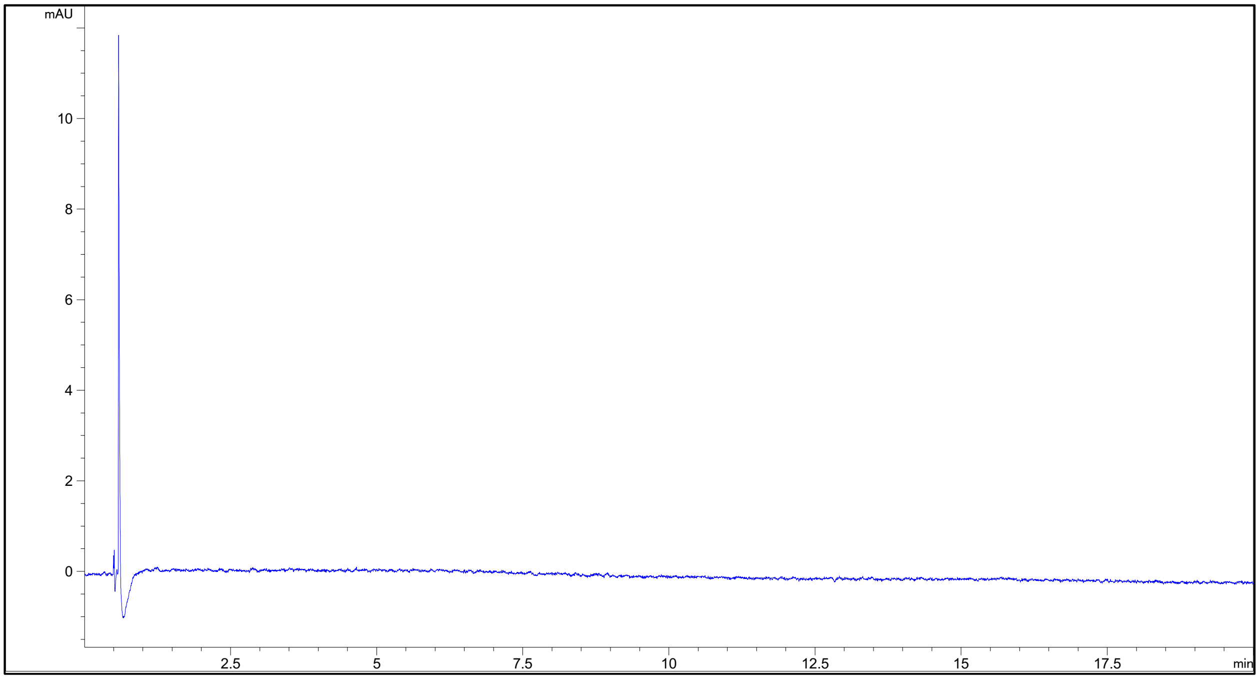

minutes

R. hirsuto-glandulosa leaf

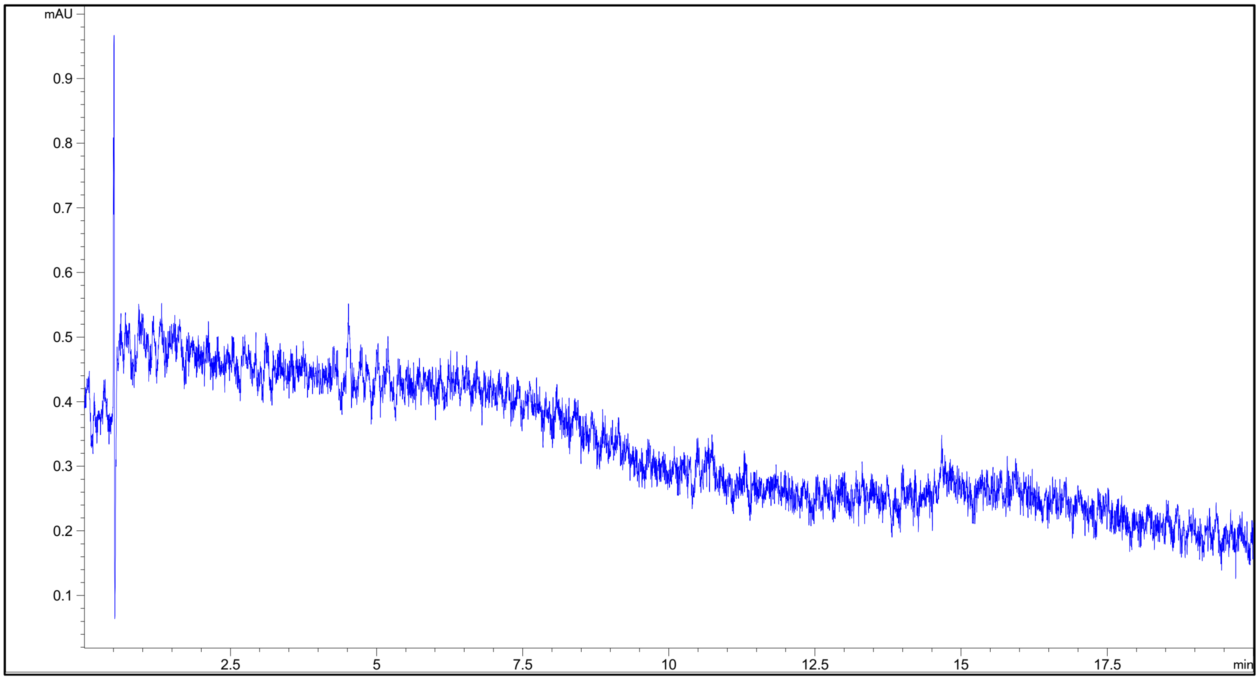

minutes

R. fulgida leaf

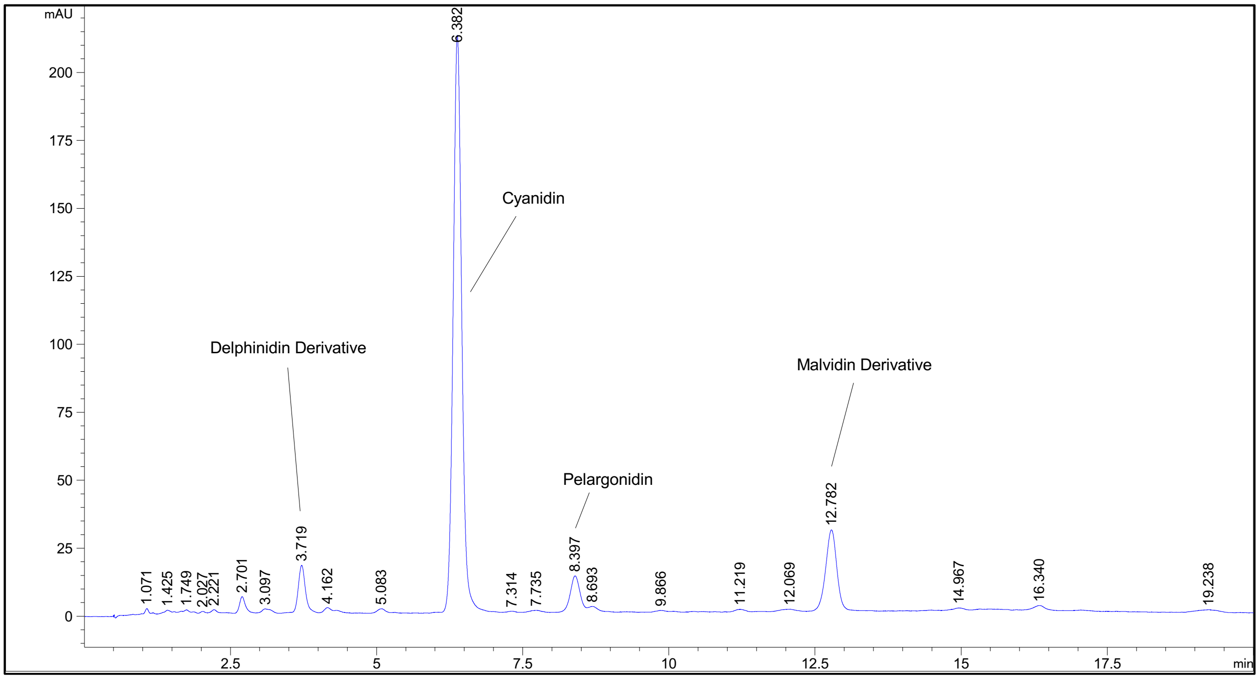

minutes

R. simplex leaf

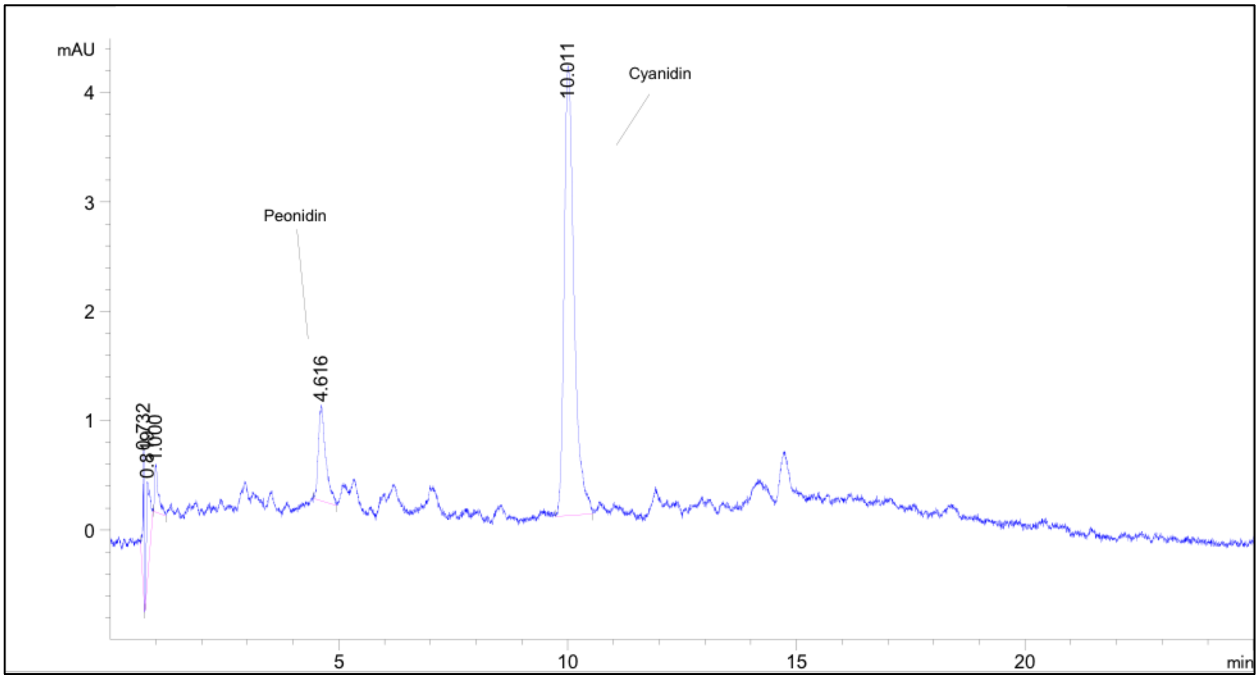

Supplement: Supplementary file 16 — Additional file 16. Table S2. Custom primers used in the qRT-PCR analysis. [file 12862_2021_1955_MOESM16_ESM.pdf]
